# Patient‐Derived Tumor Organoids Combined with Function‐Associated ScRNA‐Seq for Dissecting the Local Immune Response of Lung Cancer

**DOI:** 10.1002/advs.202400185

**Published:** 2024-06-19

**Authors:** Chang Liu, Kaiyi Li, Xizhao Sui, Tian Zhao, Ting Zhang, Zhongyao Chen, Hainan Wu, Chao Li, Hao Li, Fan Yang, Zhidong Liu, You‐Yong Lu, Jun Wang, Xiaofang Chen, Peng Liu

**Affiliations:** ^1^ School of Biomedical Engineering Tsinghua University Beijing 100084 China; ^2^ Department of Thoracic Surgery People's Hospital Peking University Beijing 100034 China; ^3^ Beijing Advanced Innovation Centre for Biomedical Engineering Key Laboratory for Biomechanics and Mechanobiology of Ministry of Education School of Biological Science and Medical Engineering Beihang University Beijing 100083 China; ^4^ Beijing Chest Hospital Capital Medical University & Beijing Tuberculosis and Thoracic Tumor Research Institute Beijing 101125 China; ^5^ Laboratory of Molecular Oncology Key Laboratory of Carcinogenesis and Translational Research (Ministry of Education) School of Oncology Beijing Cancer Hospital and Institute Peking University Beijing 100142 China; ^6^ Changping Laboratory Beijing 102299 China

**Keywords:** function‐associated single‐cell RNA sequencing, immune checkpoint blockade, local tumor immune microenvironment, primary lung cancer organoid, tumor micro‐niche

## Abstract

In vitro models coupled with multimodal approaches are needed to dissect the dynamic response of local tumor immune microenvironment (TIME) to immunotherapy. Here the patient‐derived primary lung cancer organoids (pLCOs) are generated by isolating tumor cell clusters, including the infiltrated immune cells. A function‐associated single‐cell RNA sequencing (FascRNA‐seq) platform allowing both phenotypic evaluation and scRNA‐seq at single‐organoid level is developed to dissect the TIME of individual pLCOs. The analysis of 171 individual pLCOs derived from seven patients reveals that pLCOs retain the TIME heterogeneity in the parenchyma of parental tumor tissues, providing models with identical genetic background but various TIME. Linking the scRNA‐seq data of individual pLCOs with their responses to anti‐PD‐1 (αPD‐1) immune checkpoint blockade (ICB) allows to confirm the central role of CD8^+^ T cells in anti‐tumor immunity, to identify potential tumor‐reactive T cells with a set of 10 genes, and to unravel the factors regulating T cell activity, including *CD99* gene. In summary, the study constructs a joint phenotypic and transcriptomic FascRNA‐seq platform to dissect the dynamic response of local TIME under ICB treatment, providing a promising approach to evaluate novel immunotherapies and to understand the underlying molecular mechanisms.

## Graphical abstract

In vitro models with tumor immune microenvironment (TIME) are crucial for understanding tumor immunotherapy. By developing function‐associated single‐cell RNA sequencing (FascRNA‐seq) platform, researchers utilized 171 individual primary lung cancer organoids (pLCOs) from seven patients, thus dissected the tumor immunity involving CD8^+^ T cells and macrophages under anti‐PD‐1 treatment, and identified *CD99* as a potential regulator of T cell activity.

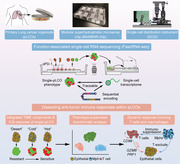

## 1 Introduction

Accurate in vitro models of the patient specific tumor immune microenvironment (TIME) and robust analysis techniques enabling in‐depth dissection of these models are indispensable for the development of next generation immunotherapy. Imaging‐based and sequencing‐based analysis of the surgically dissected patient tissues has enabled the deconvolution of not only the cell types that constitute the TIME but also their cell‐state heterogeneity.^[^ ^1^, ^2^, ^3^, ^4^ ^]^ However, they can only provide the snapshots rather than the dynamics of TIME, i.e., the subtle changes of TIME in response to treatments. Simplified in vitro models representing the in vivo tumor micro‐niche, retaining the local TIME, and facilitating the modulation of drug treatment are desired. Patient‐derived organoids (PDOs) have been widely accepted as a valuable in vitro tumor model that faithfully recapitulates the histological and molecular characteristics of original tumors.^[^ ^5^, ^6^, ^7^, ^8^, ^9^ ^]^ Depending on the methods for organoid generation and the period of in vitro culture, tumor organoids can preserve the immune components of the parental tissues.^[^ ^10^, ^11^ ^]^ For example, the PDOs established using mechanical crushing methods and cultured either in a gel matrix^[^ ^12^ ^]^ or an air‐liquid interface^[^ ^13^ ^]^ contained tumor infiltrating lymphocytes and responded to immune checkpoint blockade (ICB).^[^ ^12^, ^13^ ^]^ However, whether they recapitulate the inter‐ and intra‐ patient heterogeneity of local TIME remains to be proved. More importantly, limited by the number of cells, current analysis methods for PDOs are mainly phenotypic‐ or imaging‐based, impeding the fully dissection and wide application of PDOs as a model of patient TIME.

Single‐cell RNA sequencing (scRNA‐seq) is an indispensable tool to dissect the cellular diversity within a complex biological system. However, current sequencing‐based techniques are not applicable for deciphering the TIME in an organoid owing to the disruption of the spatial distribution of immune cells. For example, the commonly used scRNA‐seq techniques, such as 10x Chromium, stochastically capture and encode single cells, leading to the requirement of a minimum of 10^4^–10^5^ starting cells with a considerable cell loss.^[^ ^14^ ^]^ The efficient processing of every single cell from each individual organoid remains challenging. Although ample single cells can be digested from PDOs and pooled for processing, the link of single cells with their specific microenvironment within individual PDO will be disrupted (i.e., immune cells in individual organoids are not traceable). Actually, the retrospection of single cells to their parental organoids is extremely important, as it would enable the single‐cell data analysis under the supervision of phenotypic responses of organoids.

**Figure 1:**
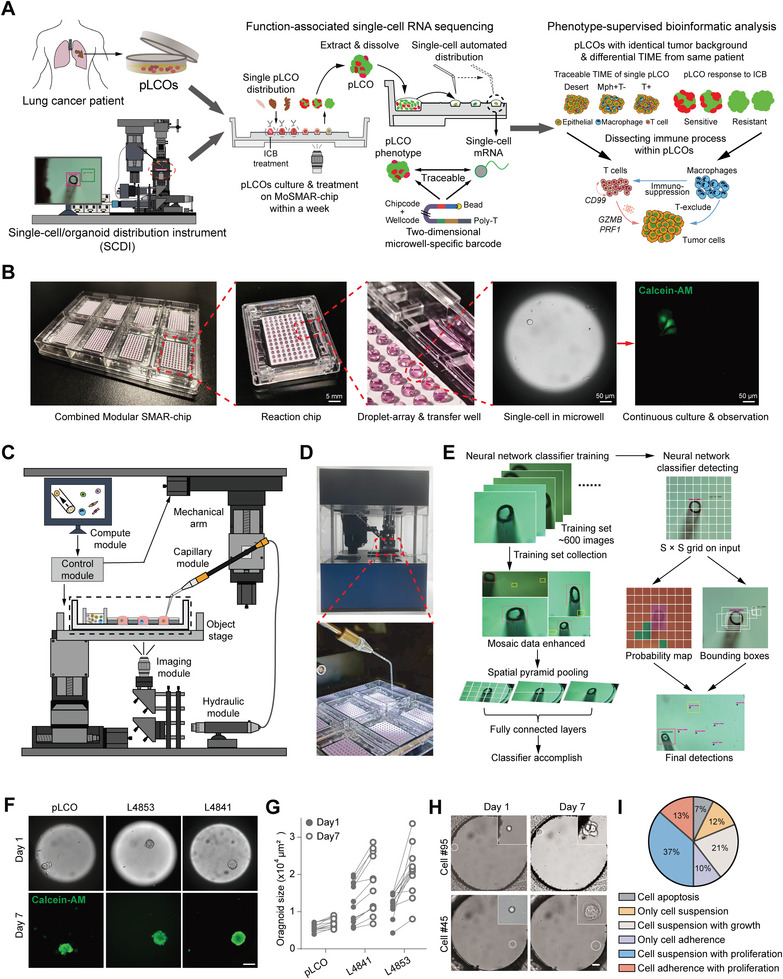
The single‐cell and single‐organoid distribution platform. A) Schematic design of the single‐pLCO analysis using FascRNA‐seq. After patient‐derived lung cancer organoids were generated, single pLCOs were distributed into MoSMAR‐chip microwells followed αPD‐1 or IgG treatment within a week. Individual pLCO was stained with Calcein‐AM/PI for phenotypic evaluation, then digested into single cells for FascRNA‐seq. The downstream bioinformatics analysis was conducted with phenotype supervision for immune process interpretation. B) Images of the MoSMAR‐chip and single‐cell culture within microwells. Eight reaction chips were installed on the chip frame and each reaction chip consists of 12×8 microwells, with corresponding droplets for cell culture, staining and observation. C) Schematic diagram and D) images of the automated single‐cell/organoid distribution instrument, consisting of the imaging, computing, motion control, mechanical arm, object stage, hydraulic and capillary modules. E) Schematic diagram of process for establishing the YOLO neural network for single cell/organoid recognition. The network was trained with ≈600 collected images to form the classifier, then the neural network classifier was applied to identify capillary, single cells, and single organoids. F) Images of single organoids distributed in the microwells and traced for seven days. At day‐7, the organoids were stained with Calcein‐AM to confirm their viability. Scale bar: 100 µm. G) Size of the traced single organoids at day‐1 and day‐7 post distribution (*n* = 36). H) Images of single cells disgested from L4853 organoids and cultured in the microwells of a MoSMAR‐chip. The circle indicates the single cell which is shown by the enlarged image at the upper right corner. Scale bar: 50 µm. I) Various circumstances of L4853‐derived single‐cell culture after seven days on a MoSMAR‐chip (*n* = 96).

In order to probe the immune microenvironment in local tumor micro‐niches, we established primary lung cancer organoids (pLCOs) from dissected tumor tissues of lung cancer patients. Owing to the nature of the mechanical processing methods, clusters of tumor cells were isolated and the infiltrating immune cells were retained. We proved that the pLCOs represent the general and patient‐specific features of local TIME in tumor parenchyma. To perform single‐cell analysis on individual pLCOs, we developed the function‐associated scRNA‐seq (FascRNA‐seq) platform, with which the phenotypic changes of pLCOs under ICB treatment were evaluated, and in the meanwhile, the transcriptomes of all the cells from each pLCO were sequenced and correlated to the phenotypic data (**Figure** **1**A). Subsequent bioinformatic analysis was conducted with phenotype supervision, which certified that the anti‐PD‐1 (αPD‐1) induced cytotoxic effect was mediated by CD8^+^ T cells with patient‐specific activation patterns. Our methods can be used for characterizing the local tumor micro‐niche, identifying the tumor‐reactive T cells, and potentially facilitating the development of novel immunotherapy strategies.

## 2 Results

### 2.1 Automated Single Cell and Organoid Distributions

The cornerstones of the FascRNA‐seq system are the modular superhydrophobic microarray chip (MoSMAR‐chip), which is the reactor for tumor organoid culture, drug sensitivity test, and scRNA‐seq sample preparation (Figure 1B; Figure S1, Supporting Information), and the automated single‐cell/organoid distribution instrument (SCDI), which automatically delivers single cells/organoids to each microwell of MoSMAR‐chip (Figure 1C–E; Figure S2, Supporting Information). The MoSMAR‐chip fabricated by injection molding with polystyrene consists of eight 96‐microwell reaction chips assembled in a chip frame, achieving a higher throughput of 768 for FascRNA‐seq. The reaction chip is designed to accommodate a transfer coverslip (Figure S1E, Supporting Information), which can deliver reagents to each microwell by a “spot‐cover” method. The droplet‐to‐droplet contact between the reaction chip and transfer coverslip is realized due to the home‐made superhydrophobic paint on the top surfaces of chips.^[^ ^15^ ^]^ In the reaction chip, the 12×8 microwell array is surrounded by eight large transfer wells (Figure 1B), in which cell suspension can be preloaded or organoids can be digested to single cells for the subsequent single‐cell distribution with minimum transfer distances.

We developed the SCDI employing a microneedle‐based liquid handling robotic under the control of an AI‐assisted target detection software. The image recognition employing the YOLO neural network^[^ ^16^ ^]^ as computing framework was trained with a dataset of ∼600 images, achieving the real‐time recognition and positioning of microneedle and single cells (Figure 1E; Figure S2A, Supporting Information). The cell distribution can be operated in different modes for rare (i.e., cells isolated from an organoid) or sufficient cell samples (i.e., digested cell line samples) (Figure S2B, Supporting Information) and adapted to capillaries with different diameters for samples with different sizes (i.e., single cells and organoids). The distribution accuracy of 91.3% and 94.6% were achieved for rare single‐cell and single‐organoid samples, respectively (Figure S2C–F; Movies S1 and S2, Supporting Information). The compact layout and barrier‐free interface between microwells of MoSMAR‐chip shortened the shuttle path of microneedle, thus enabled more efficient operation compared to multi‐well plates (Movie S3, Supporting Information) with a distribution rate of 2.5 s per cell on chips (Figure S2F, Supporting Information). We delivered two organoid lines generated in our previous study and one primary organoid at day‐1 post sample collection on the MoSMAR‐chip. After one‐week culture, all the organoids had proliferated compared to day‐1 (Figure 1F,G). In addition, single cells digested from organoid line L4853^[^ ^15^ ^]^ also showed good viability (Figure 1H,I), indicating the good biocompatibility of the distributing process.

### 2.2 Function‐Associated Single‐Cell RNA Sequencing

**Figure 2:**
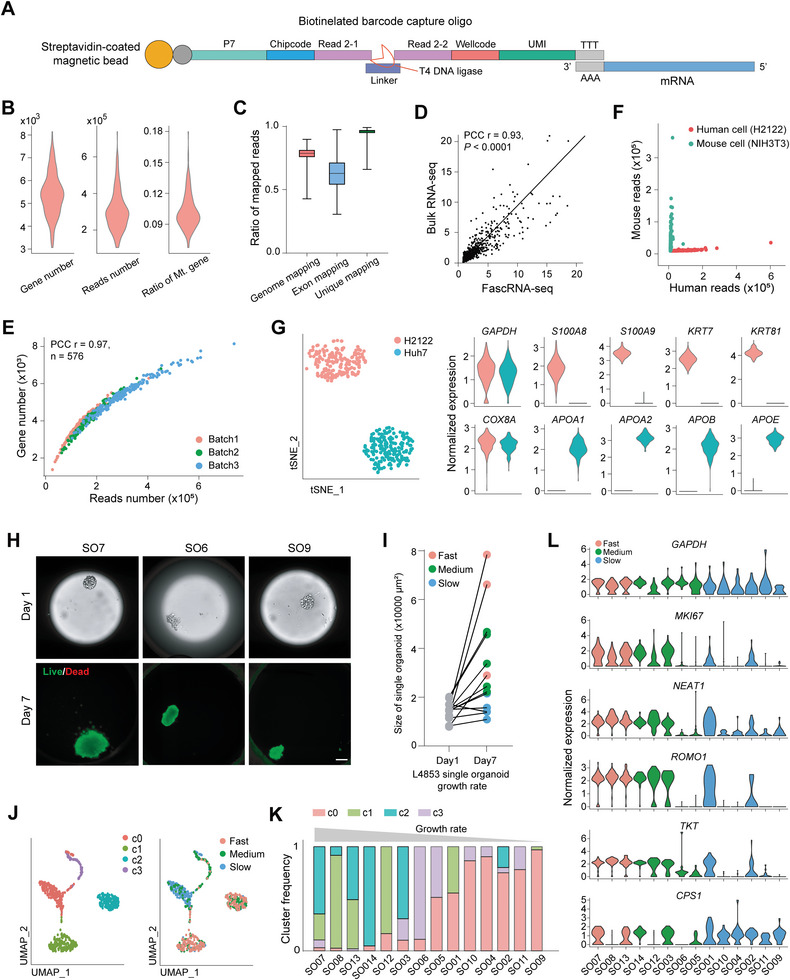
Verification of FascRNA‐seq. A) Structure of the 3′ mRNA capture oligo for FascRNA‐seq. B) Violin plots of the number of genes (average 5380), mapped reads (average 30522), and ratio of mitochondria‐related genes (Mt. gene) detected by FascRNA‐seq for 192 H2122 cells. C) Ratio of mapped reads for 192 H2122 cells. D) Correlation between the average of 192 single‐cell transcriptomes and bulk RNA‐seq data. The top 1000 genes detected in FascRNA‐seq were compared. E) Numbers of reads and genes detected in single cells for three repeats of FascRNA‐seq (*n* = 576). F) Numbers of mouse reads and human reads detected in each microwell of a MoSMAR‐chip which is distributed with single mouse cells (NIH3T3) and human cells (H2122) in alternate lines (*n* = 192). G) Cell type profiling of the H2122 and Huh7 cell lines using FascRNA‐seq. Left: tSNE visualization of scRNA‐seq data from two types of cells. Right: violin plots showing the expression levels of signature genes for lung (H2122) and liver (Huh7) cells (*n* = 384). H) Representative images of 14 L4853 organoids cultured on chip for seven days. On day‐7, the organoids were stained with Calein‐AM/PI to verify their viability. Scale bar: 100 µm. I) Quantification of the growth rates (GR) of 14 organoids. Individual organoids were categorized into three groups according to their GR (Fast, GR ≥ 3; Medium, 1.5 < GR < 3; Slow, GR ≤ 1.5). J) UMAP visualization of 672 single cells derived from 14 L4853 organoids. Cells were color labeled with the unsupervised clusters (left) or the categories according to GR (right) of the parental organoids. K) Proportions of cells from four clusters in the 14 organoids. L) Average expression of selected reference gene (*GAPDH*), typical proliferation gene (*MKI67*) and malignancy related genes in 14 organoids. Individual organoids are color labeled according to their groups of GR. Note the upregulated expression of these genes in fast growing organoids.

To perform scRNA‐seq on single cells digested form organoids, we upgraded our previously developed Group‐seq methodology^[^ ^17^ ^]^ to the single‐cell level with a higher throughput with MoSMAR‐chip. Since we need to record the barcode in each microwell to trace single cells, we employed a ligation‐based 2D barcode synthesis method to lower the costs of the long capture oligos that attach to magnetic beads for mRNA capture (**Figure** **2**A). The structure of our barcode consisted of the chipcode on P1 and wellcode on P2, consistent with the form of MoSMAR‐chip. To validate the performance of FascRNA‐seq, 192 human lung cancer cells (H2122) were distributed and sequenced. On average, more than 30 000 transcripts corresponding to 5820 genes were detected from each cell, while the ratio of mitochondria genes is less than 11% (Figure 2B), indicating the good library quality. The sequencing data had a mapping rate of ≈78%, an exon mapping rate of ≈63%, and a unique mapping rate of ≈96% (Figure 2C), comparable to previously reported magnetic bead‐based scRNA‐seq methods, such as seq‐well^[^ ^18^ ^]^ and drop‐seq.^[^ ^19^ ^]^ Besides, the expression features of FascRNA‐seq correlated well with bulk‐seq results for identical batch of cells (Pearson correlation coefficient (PCC) r = 0.93, Figure 2D). Furthermore, the repeatability of FascRNA‐seq was demonstrated by high correlation among three batches of experiments (PCC r = 0.97, Figure 2E). We also tested the inter‐microwell cross contamination by delivering human cells (H2122) and mouse cells (NIH3T3) into alternate lines on the chip and found more than 99% of the 192 microwells were free of genes from the other species (Figure 2F). At last, scRNA‐seq of the mixture of H2122 and Huh7, a liver cancer cell line, revealed two separate groups featured with expression of marker genes of lung and liver cells, respectively (Figure 2G). These data demonstrated the reliability of our MoSMAR‐chip based FascRNA‐seq.

Next, we went through the whole FascRNA‐seq process (Figure S3A, Supporting Information) using organoid line L4853.^[^ ^15^ ^]^ A total of 14 individual organoids were seeded on the chip and traced for a week (Figure 2H). The growth rates (GR: area of an organoid at day‐7 divided by area at day‐1) of these organoids varied from 1.04 to 5.29 (Figure 2I). Then, these 14 organoids were picked, digested into single cells (672 cells in total) for FascRNA‐seq. Unlike droplet‐based platform where a single cell cannot be traced back to the parental organoids, the FascRNA‐seq system can identify all the single cells from each individual organoid. Downstream scRNA‐seq analysis of the 672 cells from 14 organoids resulted in four major clusters, cluster 0 (c0) and c3 mainly consist of cells from slowly growing organoids while the majority of cells in c1 and c2 were from organoids with high growth rates (Figure 2J,K). Consistent with the phenotypic data, c1 and c2 had higher expression of genes related to proliferation and malignancy, including *ROMO1, MKI67, NEAT1*, and *TKT* (Figure 2L; Figure S3B, Supporting Information). Pseudotime analysis of the potential developmental trajectories indicated that these genes were upregulated along the inferred developmental trajectory (Figure S3C, Supporting Information), suggesting the trend toward increased malignancy during the long‐term culture.

### 2.3 pLCOs Retain the Local Immune Microenvironment in Tumor Parenchyma

**Figure 3:**
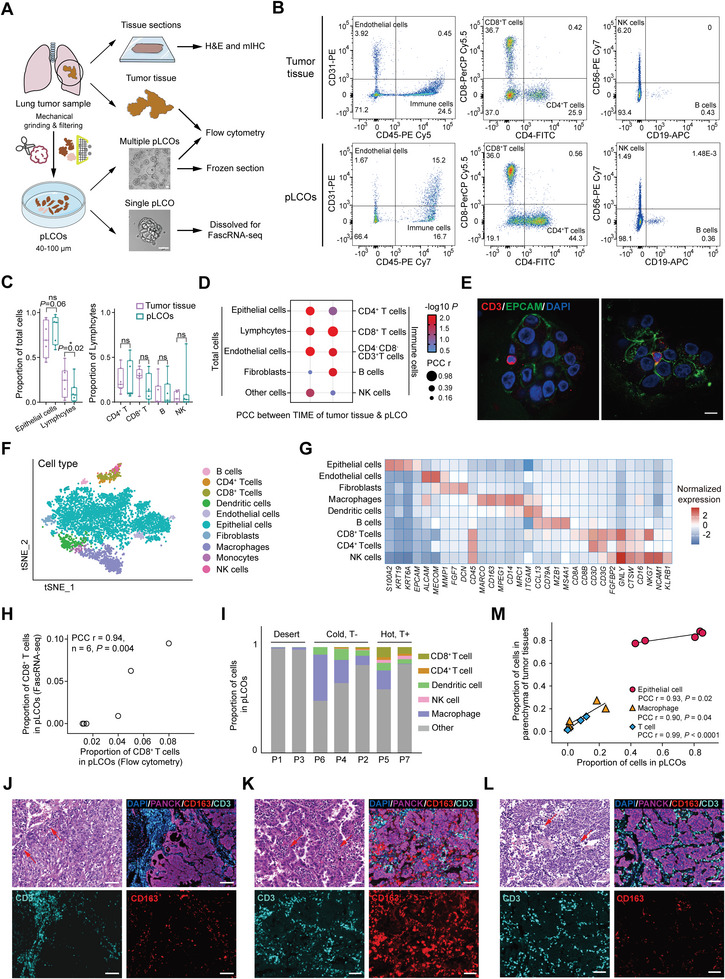
Characterization of primary lung cancer organoids. A) Scheme for pLCO generation and characterization. The pLCOs were generated by mechanical grinding and filtering from lung cancer samples, characterized mainly by flow cytometry and FascRNA‐seq. Scale bars: 50 µm. B) Flow cytometry results from the tumor tissue and pLCOs paired sample, showing the presence of immune components in pLCOs, including T cells. C) Proportions of epithelial cells, lymphocytes, and subtypes of lymphocytes in pLCOs along with the parental tumor tissues detected by flow cytometry (paired student's *t* tests, *n* = 7). D) Pearson correlation coefficient between the major TIME components of pLCOs and parental tumor tissues detected by flow cytometry (*n* = 7). E) Immunofluorescent staining images of the cryosection of pLCOs. Note the presence of CD3^+^ T cells which is in direct contact with the EpCAM^+^ epithelial cells. Scale bar: 10 µm. F) tSNE visualization of the single‐cell landscape of ≈6000 cells from 171 pLCOs. The single cells were color labeled with the annotated cell types. G) Average expression levels of marker genes for the annotated cell types. H) PCC analysis between the flow cytometry and FascRNA‐seq results on the proportion of CD8^+^ T cells in pLCOs derived from different patient samples (*n* = 6). I) Proportions of the indicated immune cells in pLCOs derived from seven lung cancer samples. Samples are categorized into three types according to the frequencies of the immune cells (desert, no immune cells; cold, few T cells; hot, abundant T cells). J–L) H&E and mIHC images of the sections from tumor tissues P3 (J), P5 (K), and P7 (L). The mIHC of tumor tissue sections were stained with CD3, CD163, and PanCK to detect the T cells, macrophages, and cancer cells, respectively. Scale bars: 100 µm. M) PCC analysis revealed significant correlation of cell proportions between pLCOs and the parenchyma region of tissue sections (*n* = 5).

In order to retain the TIME of tumor parenchyma in pLCOs, we employed a digestion‐free sample processing method developed previously,^[^ ^15^] i.e., collecting the 40–100 µm cell aggregates produced by mechanically grinding tumor tissues. Unlike the enzymatic digestion, the cell‐cell junctions were not interrupted during the processing (**Figure** **3**A). We have proved the enrichment of cancer cells and the exclusion of stromal cells, since the large extracellular matrix (ECM) fibers abundant in stroma cannot be broken by gentle grinding. Consistently, more organoids were generated from late‐stage samples which usually had higher contents of neoplastic cell.^[^ ^15^ ^]^ Therefore, we speculate that the pLCOs generated by this method retain the local micro‐niche in tumor parenchyma, including the TIME.

To prove this speculation, we first digested the pLCOs to single cells for flow cytometry analysis. Lymphocytes, including T (CD4^+^, CD8^+^), B (CD19^+^), and NK (CD56^+^) cells, were detected in pLCOs (Figure 3B). Based on this, we quantified the components of TIME in seven patients‐derived pLCOs along with their parental tumor tissues (Figure 3C). Although the proportions of lymphocytes in pLCOs were significantly lower than those in parental tumor tissues, probably due to the existence of blood vessels and tertiary lymph structures in the stroma, the proportions of T, B, and NK cells in pLCOs showed no significant difference from parental tumor tissues. Moreover, we conducted correlation analysis on the proportion of major TIME components between pLCOs and parental tumor tissues (Figure 3D), the high correlations were illustrated across epithelial cells (ECs), lymphocytes, endothelial cells, and subtypes (T and B cells) of lymphocytes, further demonstrated the characteristics of TIME in pLCOs reflected the heterogeneity among patients. Next, the immunofluorescent staining results of frozen section further demonstrated the presence of T cells in pLCOs, in contact with surrounding EpCAM expressing cancer cells (Figure 3E). Previous reports as well as our other studies indicated that the number of immune cells in tumor organoids decreased gradually with the passaging,^[^ ^13^ ^]^ so the experiments were performed within a week since formation of pLCOs.

Next, FascRNA‐seq was performed on individual pLCOs to probe the responses to ICB and the single‐cell transcription profiles. A total of 171 pLCOs generated from seven patient samples were distributed on the MoSMAR‐chip, treated with either αPD‐1 antibody or control IgG for a week, and finally digested to single cells for FascRNA‐seq. The corresponding scRNA‐seq data derived from 171 individual pLCOs was displayed as a landscape of ≈6000 cells under tSNE view (Figure 3F; Figure S4A, Supporting Information). The expression levels of marker genes were in good agreement with the annotated cell types (Figure 3G). In addition, the proportion of CD8^+^ T cells derived from FascRNA‐seq and flow cytometry analysis showed a good correlation, confirming the accuracy of annotation (Figure 3H). Consistent with the epithelial nature of the pLCOs,^[^ ^20^ ^]^ the majority (64.3%) of pLCOs were epithelial and the copy number variations (CNVs) analysis revealed that most of the cells were malignant with genetic mutations (Figure S4B,C, Supporting Information). Importantly, 25.3% of sequenced cells were immune cells, including both myeloid and lymphoid cells. Macrophages (Mphs), dendritic cells (DCs), T cells, NK cells, monocytes, and B cells were in existence of TIME and made up 13.6%, 5.2%, 4.2%, 1.1%, 0.66%, and 0.58% of total cells, respectively. Notably, pLCOs derived from seven patients represent three major classes of TIME.^[^ ^21^ ^]^ Patient 1 (P1) and P3 pLCOs showed the feature of immune “desert” with rare immune cells. P2, P4, and P6 pLCOs represented T cell excluded “cold tumor”. P5 and P7 were “hot tumor” with considerable amounts of T cells (Figure 3I).

To demonstrate whether the TIME in pLCOs represent the in vivo counterparts, we performed hematoxylin and eosin (H&E) staining, along with multiplex immunohistochemical (mIHC) staining on sections of P1, P3, P4, P5, and P7 tumors where the characteristics of TIME in tumor parenchyma were consistent with the corresponding pLCOs (Figure 3J–L; Figure S4D,E, Supporting Information). It is worth noting that the stroma showed very different immune cell infiltrating conditions. For example, condensed T cells appeared in the stroma of P3 (Figure 3J) and P4 (Figure S4E, Supporting Information) while the parenchyma was T cell excluded. Therefore, we developed an imaging processing method to quantify the fluorescent signals of immune cells in the parenchyma regions (Figure S4G, Supporting Information). The signals of T, Mph, and epithelial cells were all in strong correlation with the average proportions of those in homologous pLCOs that identified through FascRNA‐seq (Figure 3M). We noticed that pLCOs of P4 can be categorized as “desert” and “cold” organoids according to the proportion of immune cells. Consistent with this observation, the mIHC results of tissue section also revealed distinct architectures of tumor parenchyma, the solid tumor with obvious macrophage infiltration and the luminal structures with few immune cells (Figure S4E,F, Supporting Information). These results demonstrated that the pLCOs well preserved the TIME of parental tumor parenchyma and the diversities among pLCOs reflected the spatial heterogeneity of tumor parenchyma.

### 2.4 Effector‐Like CD8^+^ T Cells of pLCOs Mediate the αPD‐1 Induced Anti‐Tumor Response

**Figure 4:**
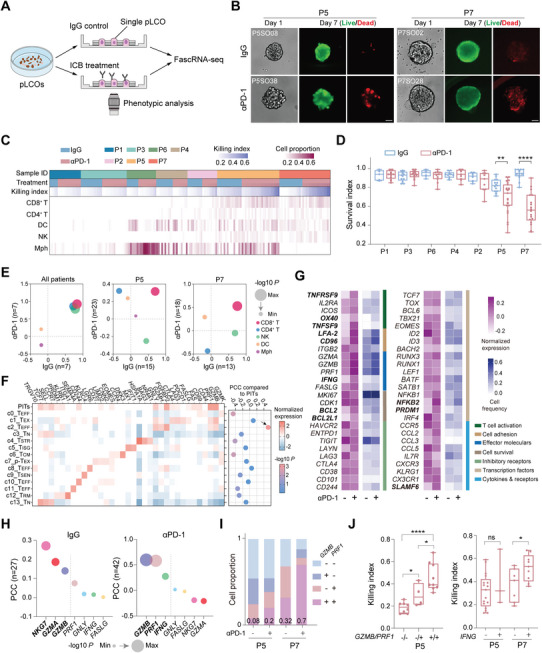
CD8^+^ T cells mediate the responses of pLCOs to αPD‐1 treatment. A) Scheme of the drug treatment experiment on individual pLCOs. B) Images of the individual pLCOs under IgG or αPD‐1 treatments. pLCOs were stained with Calcein‐AM/PI on day‐7. Scale bars: 50 µm. C) Heatmap showing the proportions of immune cells and the quantification of killing index among 171 pLCOs with or without αPD‐1 treatment. D) Comparison of the survival index of 171 individual organoids under αPD‐1 or control treatments. E) PCC analysis between immune cell proportion and the killing index. The X and Y axis show PCC for the αPD‐1 and control treatments, respectively. The data points are color labeled by the types of immune cells. The diameter of the point represents the geometric mean of the *P* value. Left: PCC analysis at the patient level where the average of organoids from the same patient sample was used as the patient data. Middle and right: PCC at the pLCO level of P5 and P7. F) Heatmap illustrating the average expression of T cell function‐associated genes in PITs of our dataset and the 14 T cell clusters reported in a pan‐cancer T cell atlas.^[^ ^23^ ^]^ PCC analysis indicating the significant correlation between expression features of PITs and c2_Teff. G) Heatmap showing the average of normalized expression of T cell function‐related genes under two treatment conditions and the proportion of cells with positive expression. The boldfaces indicate genes with significantly different levels. H) PCC analysis between the average expression level of the effector molecules in single pLCOs and the killing index. I) Quantification of the frequency of CD8^+^ T cells expressing 0, 1, or 2 of the effector molecules *GZMB* and *PRF1* in P5 (*n* = 38) and P7 (*n* = 31) pLCOs. J) Comparison of Ki with CD8^+^ T cells expression patterns of *GZMB*/*PRF1* in P5 pLCOs and *IFNG* in both P5 (*n* = 38) and P7 (*n* = 31) pLCOs.

In contrast to scRNA‐seq directly using dissected tumor tissues, in which only clinical outcomes can be referred as the overall assessment for anti‐tumor immunity, FascRNA‐seq of single pLCOs allowed us to control the treatment conditions and to evaluate the tumor killing functions of the small number of immune cells in individual organoids (**Figure** **4**A). Individual organoids showed diverse degrees of cell death, contributed by the drug treatment conditions as well as the heterogeneity in their immune microenvironments (Figure 4B,C). Significant decrease of survival index (Si: the ratio of living cells over total cells) were observed in P5 and P7 pLCOs (Figure 4D), where the number and proportion of epithelial cells were remarkably reduced, suggesting effective anti‐tumor immunity induced by αPD‐1 (Figure S5A–C, Supporting Information).

To identify the immune cell types responding to αPD‐1, we calculated the correlation between killing index (Ki = 1 – Si) and the cell proportion. At the patient level (i.e., pLCOs from the same patient were averaged), CD8^+^ T, CD4^+^ T, and NK cells all showed positive correlations with Ki under both αPD‐1 and IgG treatments. Further analysis based on single pLCOs confirmed that only CD8^+^ T showed a positive correlation with Ki consistently across patients, while CD4^+^ T and NK behaved differently in P5 and P7 pLCOs (Figure 4E). Interestingly, NK showed a positive correlation under IgG but not αPD‐1 treatment, consistent with the results of a previous report.^[^ ^22^ ^]^ Furthermore, the presence of CD8^+^ T cells significantly enhanced the Ki of pLCOs from all the samples, whereas CD4^+^ T and NK did not show such significant effect (Figure S5D–F, Supporting Information). Moreover, experiments using bulk pLCOs proved that anti‐CD8 (αCD8) antibody compromised the αPD‐1 induced anti‐tumor effect (Figure S5G,H, Supporting Information). Altogether, these data demonstrated that CD8^+^ T cells played the crucial role in αPD‐1 induced anti‐tumor immunity.

Next, we evaluated the functional status and response of these parenchyma infiltrating T cells (PITs) to αPD‐1. Displaying of the CD8^+^ T cells in tSNE showed no distinguishable clusters associated with αPD‐1 treatment or Ki of the parental organoids, whereas cells from different patients had minimum overlap (Figure S6A, Supporting Information), suggesting that the interpatient heterogeneity had a non‐negligible impact on T cell transcription status and emphasizing the need for comparisons under the same patient background. Consistent with this observation, the enrichment of inhibitory and effector molecules was not significant (Figure S6B, Supporting Information). We therefore referred to a recently published pan‐cancer T cell atlas^[^ ^23^ ^]^ to identify the status of PITs by comparing to transcription signatures of reported T cell subtypes. Based on this, PCC analysis demonstrated that PITs were significantly similar with the effector‐like cluster 2 (c2) of T cells with the lack of inhibitory receptors and the upregulated effector molecules, but not similar with the exhausted T cells (c1 and c7) (Figure 4F).

Then, we analyzed the differential expressed genes (DEGs) under two treatment conditions, and Gene ontology (GO) analysis of DEGs revealed that pathways related to T cell activation and T cell receptor signaling were enriched under αPD‐1 treatment (Figure S6C,D, Supporting Information). Consistently, genes related to T cell activation, including *OX40*, *TNFRSF9*, *CD96*, and *BCL2L1*, were significantly upregulated (Figure 4G) and T cell proliferation was promoted (Figure S6E,F, Supporting Information). The expression of co‐stimulatory receptors increased while the inhibitory receptors did not change (Figure S7A,B, Supporting Information). These results suggest that the αPD‐1 treatment activates the PITs, promotes their proliferation, but has minus effect on their exhaustion status.

We investigated the effector molecules contributing to the tumor killing function of PITs. PCC between the average expression of effector molecules in PITs and the killing index of their parental pLCOs were calculated (Figure 4H). The correlation with *NKG7* was most significant under IgG treatment, which explained the significant anti‐tumor effect from NK cells. In contrast, the top three effectors associated with αPD‐1 were *GZMB*, *PRF1*, and *IFNG* (Figure 4H–J), all of which were reported to play important roles in CD8^+^ T mediated anti‐tumor immunity.^[^ ^24^, ^25^ ^]^ The transcription level and the proportion of PITs expressing these three molecules were elevated under αPD‐1 treatment (Figure S7C,D, Supporting Information). Interestingly, the proportion of PITs co‐expressing *GZMB* and *PRF1* increased for more than two times for both P5 and P7 pLCOs (Figure 4I) and PITs expressing both molecules were correlated with higher level of Ki (Figure 4J), indicating the synergistic effect of the two molecules, consistent with previous reports.^[^ ^26^, ^27^ ^]^

### 2.5 Combining Phenotypic and Transcriptomic Data to Identify the Tumor‐Reactive T Cells

**Figure 5:**
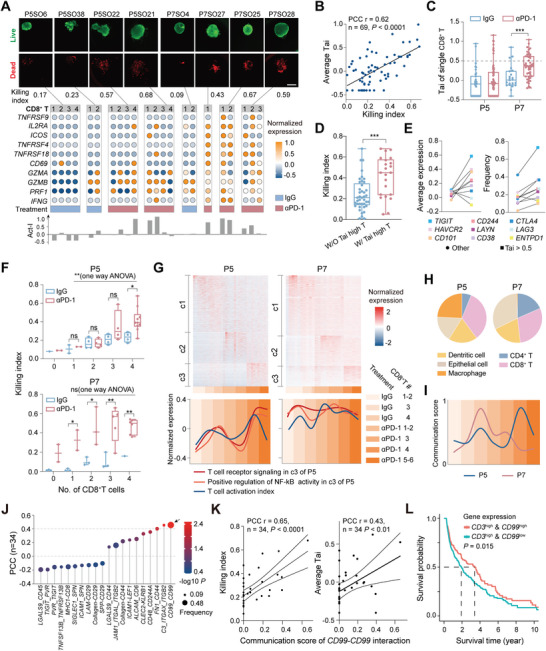
Identification of tumor‐reactive T cells and cell‐cell interactions promote T cell activation. A) Images of single pLCOs stained with Calcein‐AM/PI (top panels), bubble charts in the middle showing the expression levels of the 10 genes for calculating Tai (each column represent a single T cell), bar graph at the bottom showing Tai of each T cells. Scale bar: 100 µm. B) PCC analysis revealed significant correlation between average Tai and killing index for individual pLCOs (*n* = 69). C) Comparison of Tai for single CD8^+^ T cells under the two treatment conditions. D) Comparison of killing index for pLCOs with (w/) or without (w/o) high Tai (>0.5) cells. E) Comparison of the average expression levels (left) and frequencies (right) of the inhibitory receptors in high Tai cells and other CD8^+^ T cells. F) Comparison of cell death under the two treatment conditions in P5 (*n* = 38) and P7 (*n* = 31) pLCOs with different number of CD8^+^ T cells (one‐way ANOVA analysis for multiple groups). G) Upper panels: heatmap showing the dynamics of normalized gene expression in CD8^+^ T cells. Columns represent pLCOs ordered in treatment conditions and number of incorporated CD8^+^ T cells as indicated by the horizontal color bar at the bottom. All the genes are categorized into three groups. The c1 includes genes upregulated in IgG treated pLCOs, c2 includes genes upregulated in αPD‐1 treated pLCOs with 1–3 CD8^+^ T cells. c3 includes genes upregulated in αPD‐1 treated pLCOs with four or more CD8^+^ T cells. Lower panels: average level of all the genes from the indicated GO terms for the indicated pLCO groups. Background color represents the pLCO groups indicated on the right. Line colors indicate GO terms or the Tai. H) Pie charts showing the abundance of cell‐cell interactions received by CD8^+^ T cells and sent by different types of cells. I) Average communication scores of CD8^+^ T cell‐CD8^+^ T cell interactions for the indicated pLCO groups. Background color represents the same pLCO groups as in **(G)**. J) PCC analysis between the communication scores of ligand‐receptor pairs and killing index of P5 pLCOs. Note the homophilic interaction of *CD99* was the most significantly correlated with killing index. K) Correlation between *CD99* homophilic interaction and killing index (left) or average Tai (right) of P5 pLCOs (*n* = 34). L) The Kaplan–Meier overall survival curves of TCGA lung cancer patients (*n* = 1089), grouped by the expression level of *CD3* and *CD99* genes. *P* value was calculated by multivariate Cox regression.

Next, we tried to identify the tumor‐reactive T cells that coexist with bystanders.^[^ ^28^ ^]^ We first calculated the average expression level of genes critical for activation and function of CD8^+^ T cells in pLCOs, and analyzed the PCC with Ki (Figure S7E, Supporting Information). A total of 10 genes that had good correlation with Ki were chosen as a geneset to evaluate the functional status of CD8^+^ T cells (**Figure** **5**A), the average expression levels of which were calculated as the T cell activation index (Tai). The average Tai in individual pLCOs was significantly correlated with Ki and upregulated under αPD‐1 treatment (Figure 5B; Figure S7F, Supporting Information). Next, we calculated the Tai for all CD8^+^ T cells and identified 29 cells with Tai > 0.5 (Figure 5C). Comparison of the 29 cells with other T cells further confirmed their promoted activation level (Figure S7G, Supporting Information), while pLCOs with high Tai cells showed significantly elevated Ki (Figure 5D). Interestingly, the expression of inhibitory receptors was also promoted in high Tai cells (Figure 5E), in agreement with previous reports on the more exhausted status of tumor‐reactive T cells.^[^ ^29^, ^30^, ^31^ ^]^

### 2.6 Synergies of CD8^+^ T Cells Enhanced the Immune Activating Effect of αPD‐1

We observed that in P5 pLCOs, the significant response to αPD‐1 can only be seen in organoids with four or more CD8^+^ T cells, whereas a single CD8^+^ T cell is sufficient to produce an obvious cytotoxicity effect in P7 pLCOs (Figure 5A,F). We then analyzed whether there are gene expression patterns regulated by the number of T cells (Figure 5G). Genes were divided into three groups: upregulated in IgG treated organoids (c1), αPD‐1 treated organoids with 1–3 (c2) or more than four CD8^+^ T cells (c3). The c3 of P5 was enriched with genes correlating to T cell activation (GO terms “T cell receptor signaling” and “positive regulation of NF‐kB transcription factor activity”) while c3 of P7 was not (Table S1, Supporting Information), suggesting promoted T cell activation with increasing number in P5 organoids. To further illustrate the dynamics, the average expression levels of genes within these GO terms were plotted over CD8^+^ T cells number in single organoid. For P5 organoids, CD8^+^ T cells activation was promoted in pLCOs with increased T cells under both control and the αPD‐1 treatment, while such dynamic was not observed in P7 organoids. Consistently, Tai also increased with T cell number in P5 but not P7 organoids. These results suggest a synergistic effect between T cells which promote their activation and sensitivity to αPD‐1. Yet it seems that this synergistic effect had an impact only when T cell activation was insufficient, since this phenomenon was not observed in P7 pLCOs where T cells were activated more thoroughly (Figure 5A,F,G).

To further corroborate whether the synergistic effect between CD8^+^ T cells has a molecular base, cell‐cell interactions in individual pLCOs received by CD8^+^ T cells were elucidated through bioinformatic analysis (Figure S8A–D, Supporting Information). Consistent with the synergistic effect mentioned above, interactions between T cells were most abundant in P5 pLCOs (Figure 5H) and the communication score of CD8^+^ T‐CD8^+^ T interactions had similar dynamics as Tai (Figure 5H,I), while the antigen presentation related interactions between EC/DC and CD8^+^ T were more abundant in P7 pLCOs, which may explain the more sufficient T cell activation (Figure S8A,B,E, Supporting Information). Next, we analyzed the ligand‐receptor pairs that might contribute to the synergistic effect of CD8^+^ T cells. The PCC analysis in P5 pLCOs revealed that the homophilic interaction of *CD99* was most significantly correlated with Ki and Tai (Figure 5J,K). CD99 is a transmembrane protein broadly expressed in many cell types.^[^ ^32^ ^]^ Evidences on its costimulatory role in T cell activation has been reported.^[^ ^33^, ^34^ ^]^ Stimulation of CD99 with agonistic antibodies enhanced the expression of several T cell activation markers and sensitizes cells for activation through the CD3 signaling.^[^ ^35^ ^]^ Consistent with these reports, P5 pLCOs with T cells receiving *CD99* homophilic interactions showed significantly enhanced Ki compared to P7 pLCOs (Figure S8F,G, Supporting Information). These data suggested that the homophylic interaction of *CD99* might play a role in T cell activation in a suboptimal immune microenvironment. Moreover, our analysis on the independent lung adenocarcinoma and squamouscarcinoma cohorts (*n* = 1089) from The Cancer Genome Atlas (TCGA) further indicated that patients with high expression of *CD99* gene on T cells were associated with significant better overall survival (OS) (Figure 5L), reminding us the potential as tumor immune regulator of *CD99*.

### 2.7 Gathering of M2‐Like Macrophages Prevents the Accumulation of T Cells

**Figure 6:**
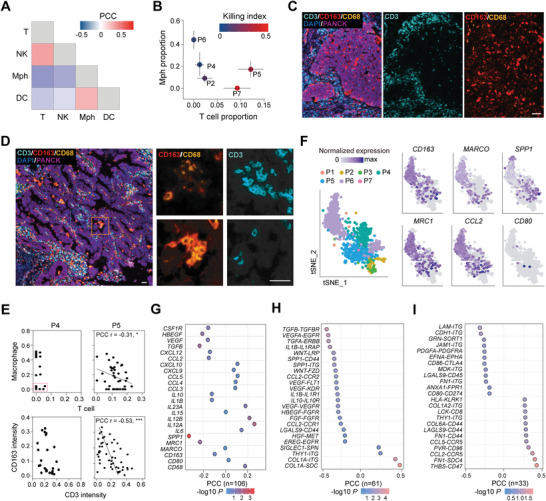
Gathering of M2‐like macrophages prevents the accumulation of CD8^+^ T cells. A) Heatmap showing PCC between the proportions of different immune cell types (*n* = 171). Note the negative correlation between Mphs and T cells. B) Dotplot showing the average proportions of T and Mph in the pLCOs from different patient samples. The lines on the dots represent the standard deviation and the color of the dots indicates the average Ki for pLCOs. C,D) mIHC images of P4 (C) and P5 (D) tumor tissue sections. Note the excluding of T cells from tumor parenchyma of P4 and the presence of both T and Mph in P5. Scale bars: 60 µm. E) Quantification of T cell and Mph proportions in individual pLCOs (upper panels, *n* = 57) and the CD3 and CD163 signals in randomly picked 0.2 mm × 0.2 mm regions of the parental tumor parenchyma (lower panels, *n* = 85). Note the similar trends of the T over Mph ratio in individual pLCOs and parenchyma regions of the same patient sample. F) tSNE visualization of Mphs from all pLCOs. Data points of each cell were labeled with patient ID or normalized expression of indicated genes. G) PCC between T cell number and average expression level of tumor immune related genes in the Mphs of pLCOs. H,I) The ligand‐receptor pairs between Mphs and ECs (H) or between Mphs and T cells (I) that had the most significant correlation with the number of T cells in pLCOs. Note the negative correlation for most of the ligand‐receptor pairs between Mphs and ECs.

pLCOs derived from patient samples varied in their immune cell components, yet such variations were not completely random but exhibited some general and patient‐specific characteristics. Among the pLCOs with immune cells, the high proportion of myeloid cells, mostly macrophages, was associated with inhibited lymphocytes accumulation and compromised response to αPD‐1 (**Figure** **6**A,B). The “repellency” of macrophages to T cells was also observed on tissue sections, as quantification of macrophage (CD163) and T cell (CD3) signals in tumor parenchyma demonstrated their negative correlation (Figure 6C–E).

Displaying the transcriptome data of macrophages in tSNE revealed obvious M2 signatures, including *CD163*, *MRC1*, and *MARCO*, while the M1 signatures such as *CD80* were indistinctive (Figure 6F). Consistent with the transcriptome data, mIHC results of tissue sections also demonstrated M2‐like tumor‐associated macrophages (TAMs) as the majority (CD163^+^ TAMs dominant). Furthermore, TAMs in P4 showed more M2‐like polarity as indicated by the ratio of CD163 over CD68 signals, in agreement with the more complete T cell elimination (Figure 6C–E). PCC analysis revealed that the expression levels of M2 signatures, epithelial‐mesenchymal transition (EMT) related genes, angiogenesis‐promoting genes (*VEGF*, *TGFB*), immune suppressive cytokines (*IL‐10*, *IL‐1B*), and monocyte recruitment chemokines (*CCL2*, *CXCL12*) were negatively correlated with T cell accumulation, while the proinflammatory cytokines, such as *IL‐12*, and the chemokines promoting T cell recruitment had a positive correlation with T cell accumulation (Figure 6G). Cell‐cell interaction analysis revealed that these molecules compromise T cell infiltration largely through the tissue remodeling associated interactions between macrophages and ECs (Figure 6H), while interactions between macrophages and T cells are heterogenous, exerting both positive and negative effects (Figure 6I; Figure S9A, Supporting Information).

Lastly, we displayed the cell‐cell interaction networks to show the local TIME in tumor parenchyma for individual patients. The interaction networks were more immunosuppressive in P6 and P4 compared to P5 (Figure S9B–D, Supporting Information). For P5, macrophages had mixed characteristics of M1 and M2 polarity. Correspondingly, the cell‐cell interaction networks in pLCOs varied from immunosuppressive to immune‐promoting. For example, *MHCI‐CD8* mediated antigen presentation and *CD86‐CD28* mediated co‐stimulation exist in αPD‐1 sensitive P5SO22 (Figure S9E, Supporting Information). Interestingly, we noticed that although the cell‐cell interaction networks in P6 were mostly immune inhibitory, interactions between macrophages and DCs were favorable for DC maturation, including *CD40LG‐CD40* interactions. In addition, these interactions enhanced significantly under αPD‐1 treatment (Figure S9D, Supporting Information). Accordingly, expression levels of genes related to DC maturation, including *MHCI*, *MHCII*, *CD86*, *CD40*, and *CCR7*, elevated significantly and the proportion of DCs decreased under αPD‐1 treatment (Figure S9F–G, Supporting Information).

## 3 Discussion

One of the key features of our FascRNA‐seq platform is the capacity of combining the functional phenotypic information of individual samples with their inner single‐cell transcriptomics, due to the precise single‐cell distribution and sequential microwell barcoding. FascRNA‐seq is suitable for analyzing samples containing no more than 10^4^ cells, for example individual organoids, especially when there are pivotal immune cells or stem cells with functions. Here, we evaluated responses of individual pLCOs to ICB, thus assessed the immune cells responsible for these changes according to their transcription profiles. In addition, our methods can also be used for similar analysis of normal organoids developed from stem cells.^[^ ^36^ ^]^

Unlike organoids developed from single “seed” cells, for example Lgr5^+^ intestine stem cells,^[^ ^37^ ^]^ which are composed of pure epithelia, our pLCOs are composed of tumor cells and infiltrated immune cells, owing to the digestion free sample processing method, by which clusters of tumor cells are isolated directly and the immune infiltrates are retained. Tumor is a heterogeneous tissue composed of parenchyma and stroma, both of which have a huge diversity in their immune infiltrates.^[^ ^2^, ^38^ ^]^ We demonstrated that the average proportions of immune cells in the pLCOs are consistent with the overall level of immune infiltrates in parenchyma, but different from that in the whole tumor tissue containing abundant stroma. In addition, we also proved that the differences among individual pLCOs derived from the same sample recapitulate the intra‐parenchyma heterogeneity, for example, the “desert” and “cold” pLCOs derived from P4, representing the two types of parenchyma architecture. Therefore, we conclude that pLCOs retain the local TIME in tumor parenchyma where it is derived.

As an in vitro model, pLCOs provide two advantages. First, pLCOs derived from the same tumor sample provide models with identical patient tumor background (i.e., recapitulating the fundamental features of parental tumor, such as genetic mutations and characteristics of immune infiltrates) but with slight variations in TIME (i.e., corresponding to the heterogeneity of TIME in parenchyma). Such diversities will help us define the patient‐specific factors affecting the anti‐tumor immune response, such as the synergistic effect of CD8^+^ T cells we observed in P5, which may otherwise be overwhelmed by the huge differences among patients. Second, the operability and traceability of pLCOs render the convenience to investigate immune cell dynamics under different treatment conditions, which can help us discover next‐generation immunotherapies and understand the mechanism of action more efficiently.

Previous studies using scRNA‐seq unraveled the heterogeneity of tumor infiltrating lymphocytes (TILs),^[^ ^39^, ^40^, ^41^, ^42^ ^]^ and the presence of exhausted T cells featuring with less of effector molecules and co‐expression of multiple inhibitory receptors.^[^ ^42^, ^43^ ^]^ Yet, the spatial distribution of T cells with different status are unclear. Here we observed that the parenchyma infiltrating CD8^+^ T cells in pLCOs are effector‐like rather than exhausted. Our other study on breast cancer also demonstrated that compared to those isolated from fresh tumor tissues, CD8^+^ T cells in pLCOs are less exhausted. One possible explanation to this observation is that the distribution of exhausted T cells is uneven between tumor parenchyma and stroma. The current pLCO model represents the local TIME in tumor parenchyma, but lacks the surrounding tumor stroma which contains cancer‐associated fibroblasts as well as abundant immune components, such as the tertiary lymphoid structures (TLS) and blood vessels. In the future, it will be interesting to analyze immune cells from other parts (i.e., stroma and TLS) by modifying the tissue processing method to splice the immune landscape of the whole tumor tissue, and furthermore, to elucidate the subtype and molecular features of parenchyma infiltrating CD8^+^ T cells by co‐culturing the pLCO model with peripheral blood or tumor infiltrating lymphocytes, therefore constructing more sophisticate 3D structures representing the stromal tissue in order to recurrence the in vivo anti‐tumor immunity of patients.

## 4 Conclusion

In this study, we developed a FascRNA‐seq platform consists of MoSMAR‐chip and automated single‐cell distribution instrument for single‐cell transcriptomic analysis at the single‐organoid level. Using this platform, we characterized the tumor microenvironment of 171 individual primary lung cancer organoids derived from seven patients, and demonstrated that pLCOs retained the fundamental features as well as the intra‐tumor heterogeneity of local TIME in the parenchyma of parental tumor tissues. Moreover, linking the scRNA‐seq data of individual pLCOs with their phenotypic responses to αPD‐1 treatment allowed us to comprehend that 1) the CD8^+^ T cells played pivotal roles during anti‐tumor immune response and exert tumor killing effect mainly through co‐expressing of *PRF1* and *GZMB*, 2) the synergistic effect between CD8^+^ T cells which is probably mediated by the *CD99* homophilic interactions that led to promoted T cell activation, as an alternative path besides antigen presentation activation, 3) the infiltration of M2‐like macrophages were negatively correlated with T cells infiltration, causing by cell‐cell interactions between macrophages and T cells along with epithelial cells. In the future, our phenotype assisted single‐cell transcriptome analysis may be further applied to predict patient response to immunotherapy and evaluate next generation immunotherapies.

## 5 Experimental Section

### Human Tumor Tissue Collection for Patient‐Derived Tumor Organoids

The lung cancer samples were obtained with informed consent of patients from the Department of Thoracic Surgery of Peking University People's Hospital (Table S2, Supporting Information), with approval from the ethical review boards of the Peking University People's Hospital under project number 2022PHB268‐001. The related experiments of patient‐derived tumor organoids were also approved by Medical Ethics Committee of Tsinghua University under project number 20210304. Main inclusion criteria included patients with clinically local advanced or metastatic lung cancer, aged 18 years or older, fresh tissues available through surgical resection of the lesions. Candidates were assessed to determine eligibility and informed consents were obtained before operation.

### Generation, Culture, Drug Treatment, and Digestion of Patient‐Derived Tumor Organoids

The lung cancer tissues, which had been stored in a preservation solution (DMEM/F12 containing 1% penicillin and streptomycin), were transported to the laboratory, and processed within 24 hours. Initially, the lung cancer tissues were documented, weighed, and measured for volume. Then, the tissues were sectioned into multiple 1–5 mm^3^ pieces without bias. The pieces were minced into small fragments using surgical scissors, followed by resuspension in preservation solution (10 mL). The suspension was filtered through a strainer (Falcon, 100 µm) with gentle grinding and pushing of tissue masses through the strainer using a syringe plunger (5 mL). After rinsing, any residual impurities on the membrane were discarded along with the strainer. Subsequently, the filtrate containing cell clusters and single cells was strained through a strainer (40 µm) to collect the cell clusters with sizes ranging from 40 to 100 µm. The filter membrane was separated from strainer, placed in lung cancer organoid medium (LCOM, 2 mL) (Table S3, Supporting Information), and thoroughly washed with a pipette to release cell clusters into the media. The membrane was then discarded, and samples were cultured in suspension overnight.

For culturing pLCOs in a multi‐well plate, the pLCOs in suspension were centrifuged at 500 × g for 5 min at 4 °C and then resuspended in cold growth factor‐reduced Matrigel (BD Biosciences). Subsequently, drops (60 µL) of the Matrigel cell cluster suspension were plated onto ultra‐low attachment 96‐ or 48‐well plates with a flat bottom (Corning) and allowed to solidify at 37 °C for 20 min. The seeding density was adjusted to approximately 500 organoids per well. Once the Matrigel had solidified, LCOM (100 µL) was added to each well, and the plate was transferred to a cell culture incubator (37 °C with 5% CO_2_). The LCOM medium was refreshed twice a week.

Individual tumor organoids were inoculated into the microwells of the MoSMAR‐chip using the single‐cell distribution instrument. Next, the tumor organoids were treated with Nivolumab (Selleckchem, 10 µg mL^−1^) and Human IgG4 Antibody (BioLegend, 10 µg mL^−1^). After a culture period of 5–7 days, the tumor organoids were moved one by one from the microwells to the transfer wells, where the organoids were digested into single cells by a 20 min incubation at 37 °C with TrypLE solution. Then, gentle pipetting with a 20‐µL pipette tip was performed for ≈60 times to completely digest the tumor organoids. After a settling period of 10 min, the single‐cell distribution instrument was employed to distribute these cells into microwells to form single‐cell array for subsequent FascRNA‐seq. Detailed information of tumor organoids treatment related reagents can be found in Table S4 (Supporting Information).

### Staining of Patient's Tumor Tissue Slices and Patient‐Derived Tumor Organoids in Frozen Sections

Immunohistochemistry staining was performed on paraffin slides of lung tissue with 2 µm thicknesses. The slides were initially heated at 72 °C for 30 min to ensure proper tissue adhesion. The slides were deparaffinized in xylene for 10 min and repeated three times, and rehydrated in absolute ethyl alcohol for 5 min and repeated twice, 95% ethyl alcohol for 5 min, 75% ethyl alcohol for 2 min, sequentially. Then the slides were washed with distilled water 3 times. A microwave‐oven is used for heat‐induced epitope retrieval. During epitope retrieval, the slides were immersed in boiling EDTA buffer for 15 min. Antibody diluent and block buffer (AlphaX Biotech) were used for blocking. The mIHC staining part was performed and analyzed according to a 6‐plex‐7‐color panel. All the primary antibodies (CD3, CD68, PANCK, PD‐L1, CD163, and CD56) were incubated for one hour at 37 °C. Then slides were incubated with Ploymer HRP (AlphaX Biotech) for 10 min at 37 °C. AlphaTSA Multiplex IHC Kit was used for visualization. After each cycle of staining, heat‐induced epitope retrieval was performed to remove all the antibodies including primary antibodies and secondary antibodies. The slides were counter‐stained with DAPI for 5 min and enclosed in Antifade Mounting Medium (NobleRyder). For hematoxylin‐eosin staining, the corresponding tissues were fixed in 1 mL of 4% paraformaldehyde, followed by dehydration, paraffin embedding, sectioning, and a standard H&E staining protocol. Axioscan7 (ZEISS) was used for imaging the visual capturing. Detailed information of the antibodies can be found in Table S4 (Supporting Information).

The cell proportions of tumor tissues were evaluated based on the immunohistochemistry staining images with ImageJ software. Specifically, the tumor parenchyma as image ROI was inferred according to the merged image channel, then the ROI was applied to the channel of DAPI signal and excluded the stroma region outside ROI. The threshold was applied to acquire cell nucleus signal of tumor parenchyma as base value, followed by quantification of T cells, NK cells, epithelial cells, and macrophages in the same way.

Tumor organoids were harvested from Matrigel by centrifugation at 800 rpm once they reached an approximate diameter of 100 µm. Subsequently, they were embedded in a Fibrin matrix (10 mg mL^−1^) and fixed in a 4% paraformaldehyde solution (30 min at room temperature). To facilitate cryopreservation, the fixed organoids were dehydrated overnight at 4 °C in 30% sucrose. Following the removal of sucrose, the tumor organoids were carefully embedded in cryomolds using optical coherence tomography (OCT) compound and rapidly frozen at −20 °C. Cryosections with a thickness of 25 µm were obtained and affixed onto charged slides. To ensure optimal immunostaining, the sections were permeabilized and blocked in TB buffer (TBST + 0.5% TritonX‐100 + 1% BSA) for 1 h at room temperature. Primary antibody staining was performed by incubating the slides overnight at 4 °C with EpCAM and CD3 (Abcam). Following primary antibody incubation, the slides underwent further staining with secondary antibodies, including Alexa Fluor 488‐conjugated anti‐mouse IgG, Alexa Fluor 647‐conjugated anti‐rabbit IgG, and DAPI (Invitrogen) for 1.5 h. To visualize the stained samples, fluorescence images were obtained using a Nikon A1HD25 microscope. Detailed information of antibodies can be found in Table S4 (Supporting Information).

### Quantitative Evaluation of Drug Sensitivity Response of pLCOs and Cell Proportion of Tumor Tissue

The drug sensitivity response of tumor organoid models was evaluated based on the cell viability, which was measured using Calcein‐AM/PI staining reagents. Briefly, AM solution (1 µL) and PI solution (3 µL) were added to staining buffer (100 µL). The mixture was then gently oscillated in dark for ≈1 min using an oscillator, followed by the dilution with PBS (1:30). Subsequently, the staining solution was dispensed into the microwell array of the transfer coverslip with a volume of 500 nL per well using the Echo 550 acoustic liquid handling system. Next, the reaction chip containing organoids was washed with PBS (2 mL) to replace the reagents in all the microwells. Then, the staining solution was added with transfer coverslip, followed with samples incubated for approximately 15 minutes in a light‐protected environment. The reaction chips were then retrieved and subjected to bright‐field, green, and red fluorescent imaging using an inverted fluorescence microscope (IX83, Olympus). The populations of live and dead cells were quantified by measuring the total cell area for each dye with ImageJ software. Meanwhile, the area of green signal was considered as the overall size of each tumor organoid. The response level of the tumor organoids was determined by subtracting the quantified signal of all dead cells from the quantified signal value of all live cells, which was then divided by the overall size of the tumor organoid.

### Flow Cytometry Analysis for Tumor Organoids

For flow cytometry analysis, tumor organoids were dissociated using TrypLE Express enzyme. To minimize non‐specific binding, Fc receptors (FcR) were blocked by incubating the cells with Human BD Fc Block (BD Biosciences) for 10 minutes in FACS buffer (PBS + 2% FBS). The dissociated cells were then washed with FACS buffer and stained with specific antibodies at 4 °C for 30 min. For assessing the abundance of different cell types within the tumor organoids, the antibody panel included FITC Mouse anti‐human CD326 Antibody, Pacific Blue Mouse anti‐human CD45 Antibody, PE Mouse anti‐human CD31 Antibody (BioLegend), and CoraLite Plus 647‐conjugated smooth muscle actin specific Monoclonal antibody (Proteintech). For further evaluating the frequencies of lymphocytes within the tumor organoids, the antibody panel included Pacific Blue Mouse anti‐human CD45 Antibody (BioLegend), BV510 Mouse Anti‐Human CD3, PerCP‐Cy5.5 Mouse Anti‐Human CD8, FITC Mouse Anti‐Human CD4, APC Mouse Anti‐Human CD19 and PE‐Cy7 Mouse Anti‐Human CD56 (BD Biosciences). Following the staining procedure, the cells were washed twice with FACS buffer, and a near‐infrared viability dye (Invitrogen) was added to exclude dead cells. The cell suspensions were then analyzed using the Aria III flow cytometer (BD Biosciences). Compensation beads stained with corresponding single antibodies were used to establish compensation settings. The acquired data were subsequently analyzed using FlowJo v.10.8.1 software, and only single, viable cells were included in the analysis. By examining the expression of CD326, CD31, CD45, and αSMA, epithelial cells, endothelial cells, immune cells, and fibroblasts could be identified and quantified. By examining the expression of CD45, CD3, CD8, CD4, CD19, and CD56, T cells, B cells, and NK cells could be identified and quantified. Detailed information of the antibodies can be found in Table S4 (Supporting Information).

### Design, Fabrication, and Operation of MoSMAR‐Chip

The MoSMAR‐chip was fabricated using standard injection‐molding method with polystyrene (Hochuen Technologies). Plasma cleaning was performed to make the surfaces of entire MoSMAR‐chip hydrophilic, suitable for cell culture. Next, a home‐made superhydrophobic paint (Table S5, Supporting Information) was prepared as follows: 1H, 1H, 2H, 2H‐perfluorooctyltriethoxysilane (Sigma‐Aldrich, 1 g) was first added into absolute ethanol (99 g) and mechanically stirred for two hours. Then, titanium oxide nanoparticles (TiO2, ≈60 to 200 nm, 6 g) (Sigma‐Aldrich) and P25 TiO2 (≈21 nm, 6 g) (Degussa) were added into the solution to make a paint‐like suspension, which was sonicated for 30 seconds to disperse the particles. After that, the suspension was pipetted onto the recessed top surfaces of the reaction chip and transfer coverslip, followed with air‐dried completely within 30 seconds to complete the fabrication.

For the operation of MoSMAR‐chip, the number of reaction chips assembled into the chip frame can be adjusted according to the throughput demand. The loading of reagents into the microwells can be carried out either as a whole using the immerse‐aspirate method or individually using the spot‐cover method with the transfer coverslip, which contains pre‐stored reagents formed by Echo 550 (Beckman Coulter). Cells were loaded into the microwells by random seeding or droplet rolling, and cultured in either the immersion mode or droplet mode.

### Construction and Optimization of the Automated Single‐Cell Distribution Instrument

The home‐built single‐cell distribution instrument (SCDI) consisting of an integrated microscopy module for imaging cells, a six‐axis motion and control module for operating the chip platform and the robotic arm, and a motor‐controlled hydraulic microneedle module for cell delivery. The microscopy module consists of an objective lens (Olympus), a digital camera (Daheng Optics), and a series of optical path conversion parts (Table S6, Supporting Information) to transfer images from the objective to the camera. The digital camera is linked to a PC‐based graphics card (NVIDIA) for imaging processing. The motion and control module includes a three‐axis motion platform for chip positioning and a three‐axis robotic arm for controlling the capillary movement, each of which is constructed using three 57 three‐phase stepper motors (Daheng Optics). The hydraulic module is constructed with a microinjector (Eppendorf) motorized by a 57 three‐phase stepper motor. A glass capillary electrode (B100‐75‐10, Sutter) pulled by a micropipette puller (P‐1000, Sutter) is installed onto the microinjector for extracting and releasing single cells or organoids. The synchronized control of all the movements is achieved through a stepper motor controller (Daheng Optics) that communicates with the control program via a CAN (Controller Area Network) bus.

The core of the custom‐made software is a real‐time target recognizer based on the YOLOv4 neural network framework, implemented in C++ language using the OpenCV computer vision library. To construct the training set, 600 various single‐cell images were manually captured using the single‐cell distribution instrument and labeled the regions of interest (ROIs), including the capillary microneedle tips and single cells, using the labelImg software. The training set was fed into the YOLO neural network for iterative training of the recognizer. The training process was considered complete when the loss function value fell below 0.6. The trained weight model was deposited on Github (https://github.com/JohnTerry0106/Files‐related‐to‐pLCO‐research.git).

The system optimization of the SCDI is focused on: the recognition accuracy of the neural network recognizer, the size of the neural network training set, the Ficoll ratio in cell samples, the cell density, the precision of the hydraulic‐driven single‐step movement, and the inner diameter of the capillary. The optimized parameters were determined as follows: the neural network was trained with a dataset of 600 samples; the capillary with an inner diameter of ≈100 µm was driven with a single‐step precision of 50 nL and the concentration of cell suspension was 1×10^5^ cells mL^−1^ in 5% Ficoll solution.

### Single‐Cell Transcriptome Library Construction

All the reagents used in the FascRNA‐seq protocol are listed in the Table S7 (Supporting Information), primers and barcode sequences designed for FascRNA‐seq are listed in Table S8 (Supporting Information). Briefly, the 114‐base‐long barcode that attaches to the streptavidin‐coated Dynabeads is constructed by linking two short oligos (P1 and P2) together as complete barcode with the enzymatic ligation. First, the beads and barcodes were loaded into the microwells of the transfer coverslips through Echo 550. In each microwell, barcodes (500 nL, 10 ng mL^−1^) were reacted with M270 magnetic beads (80 nL, 1:2 diluted). The loaded transfer coverslip was then stored at 4 °C until use.

Following the single‐cell loading with SCDI, the transfer coverslip containing the barcoded magnetic beads was aligned and covered onto the reaction chip. After the loading of barcoded‐beads, the reaction chip was washed with 5× RT buffer to remove any unlinked capture oligos from the microwells. Then, lysis buffer (200 µL, 200 mm Tris–HCl, 20 mm EDTA, 1% Sarkosyl, 50 mm DTT) was pipetted to cover the microwell array of reaction chip and lyse single cells in situ. The magnetic beads immediately captured the released mRNAs. Following a 15 min incubation, the beads were gently washed off from the MoSMAR‐chip with 6× SSC solution (1 mL) and collected into a 1.5‐mL centrifuge tube. Subsequently, the beads were washed three times with washing buffer (600 µL, 10 mm Tris–HCl, 15 m NaCl, 1 mm EDTA, and 0.1% Sarkosyl), and with 5× Maxima H RT buffer (50 µL). Finally, beads were resuspended in 5× Maxima H RT buffer (4 µL) and kept on ice temporarily.

The magnetic beads were loaded into reverse transcription (RT) mix (16 µL), comprising Nuclease‐Free water (10.5 µL), 10× dNTP (2 µL), template switch oligo (TSO, 2 µL, 25 µm), Reverse Transcriptase (1 µL, 200 U µL^−1^), and RNase inhibitor (0.5 µL, 40 U µL^−1^). Subsequently, the mixture was incubated at room temperature for 30 min, followed by 42 °C for 90 min to generate cDNA. The beads were then subjected to one wash with TE/SDS (20 µL, 1× TE, and 0.5% sodium dodecyl sulfate), two washes with TE/TW (20 µL, 1× TE, and 0.01% Tween 20), and resuspended in ddH2O (12 µL).

The beads were supplemented with a PCR mix comprising 2× HotStart Readymix (Kapa Biosystems, 20 µL), 5′ end biotin‐modified P7 primer (4 µL, 10 µm), and TSO primer (10 µm, 4 µL). The PCR program consisted of an initial denaturation step at 95 °C for 3 min, followed by 4 cycles of 98 °C for 20 s, 65 °C for 45 s, and 72 °C for 3 min, and subsequently, 23 cycles of 98 °C for 20 s, 67 °C for 20 s, and 72 °C for 3 min. A final extension step was 72 °C for 5 min. The PCR products were purified using AMPure XP beads (Beckman Coulter, 0.6×) and eluted into ddH2O (50 µL).

PCR products were fragmented using the M220 system (Covaris) to generate 3′‐end cDNA fragments. Then, end repair, 5′ phosphorylation, dA‐tailing, adaptor ligation, size selection, and PCR enrichment were performed sequentially using the NEBNext Ultra II DNA Library Prep Kit for Illumina. Lastly, PCR products were purified using the AMPure XP beads and assessed using Agilent Bioanalyzer 4200. The libraries were subjected to paired‐end sequencing on the HiSeq‐PE150 instrument (Illumina).

### Single‐Cell RNA‐Seq Analysis of Tumor Organoid Models

Read 1 Fastq files were aligned to an appropriate reference genome (mm10 for mouse cells and hg38 for human cells) using STAR v2.4.0a with default settings. Read 2 Fastq files were used to extract wellcodes and UMIs based on the wellcode‐UMI‐poly T pattern. The RNNS algorithm was developed for wellcode identification, which allowed up to 1‐nt mismatch with the reference. PCR duplicates were identified when the same wellcode, UMI, and gene ID were detected. The gene abundances of the samples were estimated using featureCounts (Version 1.5.0). The bioinformatic pipeline was deposited on Github with same URL above.

For the integrated scRNA‐seq dataset of patient‐derived tumor organoids, cells with >300 transcripts and <20% mitochondrial reads were retained. The filtered expression matrix was subjected to further analysis using Seurat^[^ ^44^ ^]^ (version 4.2.0). The Seurat object was normalized for the identification of variable genes and scaled. Next, the harmony algorithm^[^ ^45^ ^]^ was applied to integrate samples and eliminate batch effects of patients. tSNE dimensional reduction was performed using the first 10 principal component analysis (PCA) dimensions as input features. FindClusters() was computed at a resolution of 0.8. The pseudotime analysis for tumor organoid line dataset was performed using Monocle 3^[^ ^46^ ^]^ (version 1.2.9). The differential gene expression was analyzed using FindAllMarkers() with default parameters and the pathway enrichment analysis was conducted by clusterProfiler^[^ ^47^ ^]^ (version 4.4.4).

Cell type annotations were performed through SingleR^[^ ^48^ ^]^ (version 1.10.0) with reference datasets of HumanPrimaryCellAtlas and BlueprintEncode. The identified immune cells were further validated using five additional datasets, including ImmuneCellExpressionData, Hematopoietic, Monaco, MonacoImmuneData, and Novershtern HematopoieticData. In this workflow, the annotations were further refined by combining with the expression features of Seurat clusters to complete the identification of cell types across the dataset.

InferCNV^[^ ^49^ ^]^ (version 1.12.0) was adopted to estimate the chromosomal CNVs of epithelial cells following the standard workflow with default parameters. The inferCNV results of each cell were quantified as a CNV score. Specifically, the complete loss of copy or addition of more than one copy was counted as two points, the loss or addition of one copy was counted as one point, and the neutral situation was not counted.

The ligand‐receptor interaction based cell communication analysis of each single‐organoid level sub‐database was completed by two R packages, CellCall^[^ ^50^ ^]^ (version 0.0.0.9000) and CellChat^[^ ^51^ ^]^ (version 1.6.1). Specifically, in CellChat package related analysis, identifyOverExpressedGenes and computeCommunProb were mainly applied with default parameters to find the ligand‐receptor genes overexpressed in major cell types and identify ligand‐receptor combinations present. In CellChat package analysis, the sub‐datasets of single‐organoid were first generated as Seurat object, then the CreateObject_fromSeurat and TransCommuProfile procedure were performed with default parameters successively in order to infers intercellular communication by combining the expression of ligands/receptors and downstream TF activities for certain L‐R pairs. The calculated single‐organoid based ligand‐receptor interaction results of CellChat and CellCall were derived in order to apply further aggregated and statistic analysis.

### Statistical Analysis

GraphPad Prism 9 was used to plot and statistically analyze the data. The differences between two groups were analyzed by unpaired, two‐tailed student's *t* tests unless otherwise indicated in corresponding figure legends due to the experiment circumstances. One‐way ANOVA analysis was used to analyze significance when comparing more than two groups. The significances were denoted as * and ns, (specifically, *P < 0.05, **P < 0.01, ***P < 0.001, ****P < 0.0001, *P* value lower than 0.05 is considered significantly different). Information related to the size of data was indicated in figure panels or figure legends. For values presented as box & whiskers plots, the center lines represent the median values, the bounds of box represent the median values of the upper half and lower half, the bounds of whiskers represent the maxima and minima. Values in column bar graphs were illustrated as mean ± standard deviation (SD). Transcriptome related data process procedure was presented in experimental sections above. Flow cytometry data were analyzed with FlowJo software. Image J was used for image signal processing and quantitative statistics.

### Ethics Approval and Patient Consent Statement

There are no animal experiments performed in this study. The lung cancer tumor samples were obtained with informed consent of patients from the Department of Thoracic Surgery of Peking University People's Hospital with approval from the ethical review boards of the Peking University People's Hospital (2022PHB268‐001). The related experiments of patient‐derived tumor organoids were also approved by the Medical Ethics Committee of Tsinghua University (20210304).

## Conflict of Interest

The authors declare no conflict of interest.

## Author Contributions

C.L., K.L., and X.S. contributed equally to this work. C.L. and K.L. conducted the investigation and development of methodology. J.W., X.S., C.L., H.L., and F.Y. contributed to acquisition of clinical samples. T.Z., T.Z., Z.C., and H.W. helped with the methodology and validation. Z.L. helped with the clinical resource coordination and Y.L. provided supervision and suggestions to the study. C.L., P.L., and X.C. contributed to conceptualization and manuscript formation. P.L., X.C., and J.W. contributed to the conceptualization and supervision of this study.

## Supporting information

Supporting Information

Supporting Table S1‐S8

Supplemental Movie 1

Supplemental Movie 2

Supplemental Movie 3

## Data Availability

The data that support the findings of this study are available from the corresponding author upon reasonable request.
