# Supplementary material for: Patient‐Derived Tumor Organoids Combined with Function‐Associated ScRNA‐Seq for Dissecting the Local Immune Response of Lung Cancer
Source: Adv Sci (Weinh). 2024 Jun 19;11(31):2400185. doi: 10.1002/advs.202400185 (PMC11336893; doi:10.1002/advs.202400185)
Supplement: Supplementary file 1 — Supporting Information [file ADVS-11-2400185-s002.pdf]

ADVANCED SCIENCE
Open Access

Supporting Information

for *Adv. Sci.*, DOI 10.1002/advs.202400185

Patient-Derived Tumor Organoids Combined with Function-Associated ScRNA-Seq for Dissecting the Local Immune Response of Lung Cancer

Chang Liu, Kaiyi Li, Xizhao Sui, Tian Zhao, Ting Zhang, Zhongyao Chen, Hainan Wu, Chao Li, Hao Li, Fan Yang, Zhidong Liu, You-Yong Lu, Jun Wang, Xiaofang Chen* and Peng Liu**

Supporting Information

for Adv. Sci.

Patient-derived Tumor Organoids Combined with Function-associated ScRNA-seq for Dissecting the Local Immune Response of Lung Cancer

Chang Liu,[1#] *Kaiyi Li*,[1#] *Xizhao Sui*,[2#] *Tian Zhao*,[1] *Ting Zhang*,[3] *Zhongyao Chen*,[1] *Hainan Wu*,[3] *Chao Li*,[2] *Hao Li*,[2] *Fan Yang*,[2] *Zhidong Liu*,[5] *You-Yong Lu*,[6] *Jun Wang*,[2*] *Xiaofang Chen*,[3*] *Peng Liu*[1,4*]

# The authors contributed equally to this paper.
*Corresponding author: Peng Liu, Email: pliu@tsinghua.edu.cn
Xiaofang Chen, Email: xfchen@buaa.edu.cn
Jun Wang, Email: wangjun@pkuph.edu.cn

This supporting information file includes:

Supplementary Figures 1 to 9 (Figures S1 to S9)
Legends for Figures S1 to S9
Legends for Supplementary Tables 1 to 8 (Tables S1 to S8)
Description for Supplementary Movies 1 to 3 (Movies S1 to S3)

Other supplementary materials for this manuscript include the following:

Supplementary Tables 1 to 8 (Tables S1 to S8)
Supplementary Movies 1 to 3 (Movies S1 to S3)

Supplementary Figures

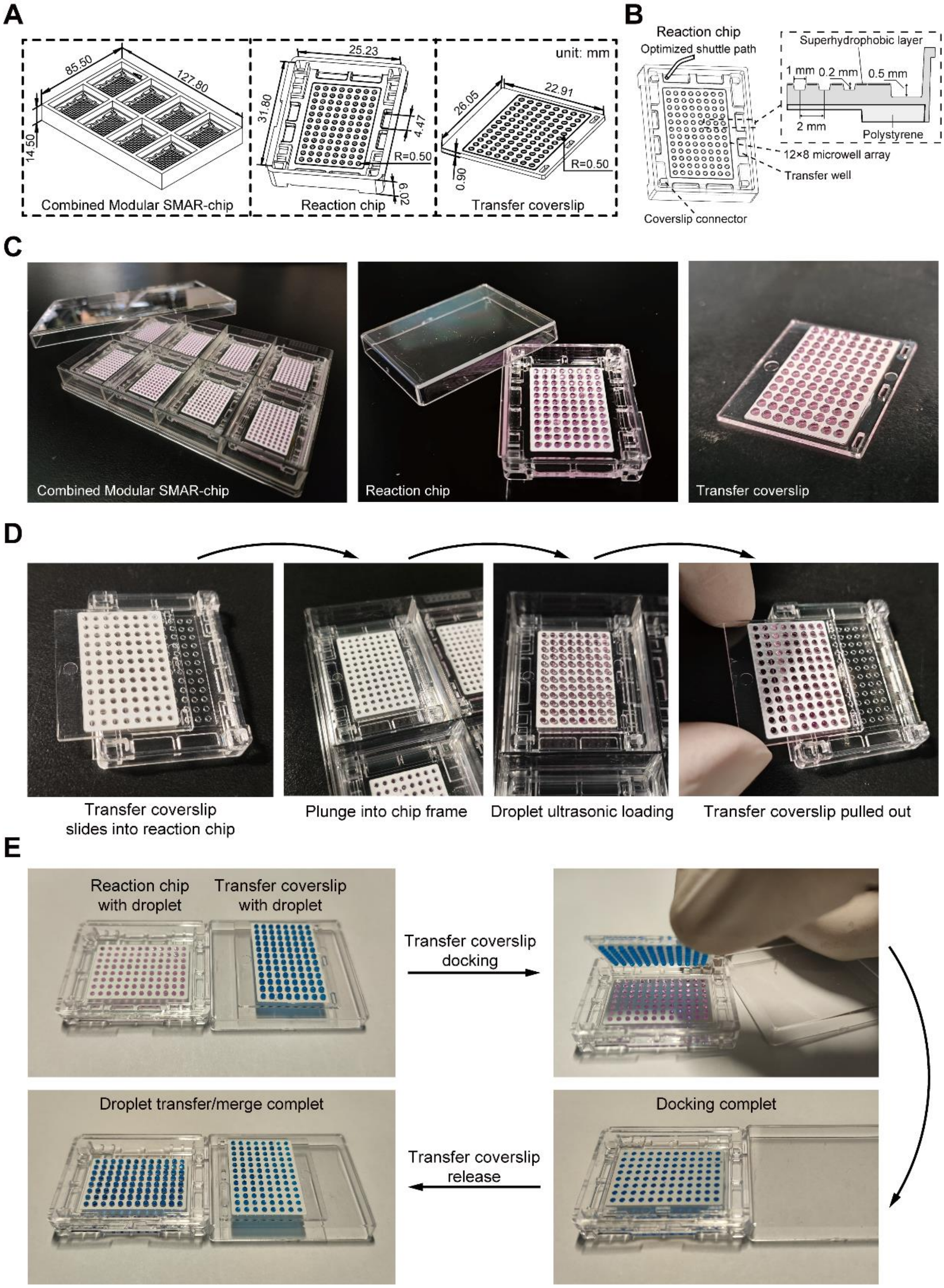

Figure S1. Architecture and operation of MoSMAR-chip. A,B and C) Schematic diagram **(A)**, section diagram **(B)** and photographs **(C)** showing the structure and dimensions of the

assembled MoSMAR-chip, reaction chip, transfer coverslip and the connectors for placing transfer coverslip. **D and E)** Images of the "spot-cover" procedure with the transfer coverslip pre-loading **(D)** and docking to the reaction chip **(E)** for reagent delivery.

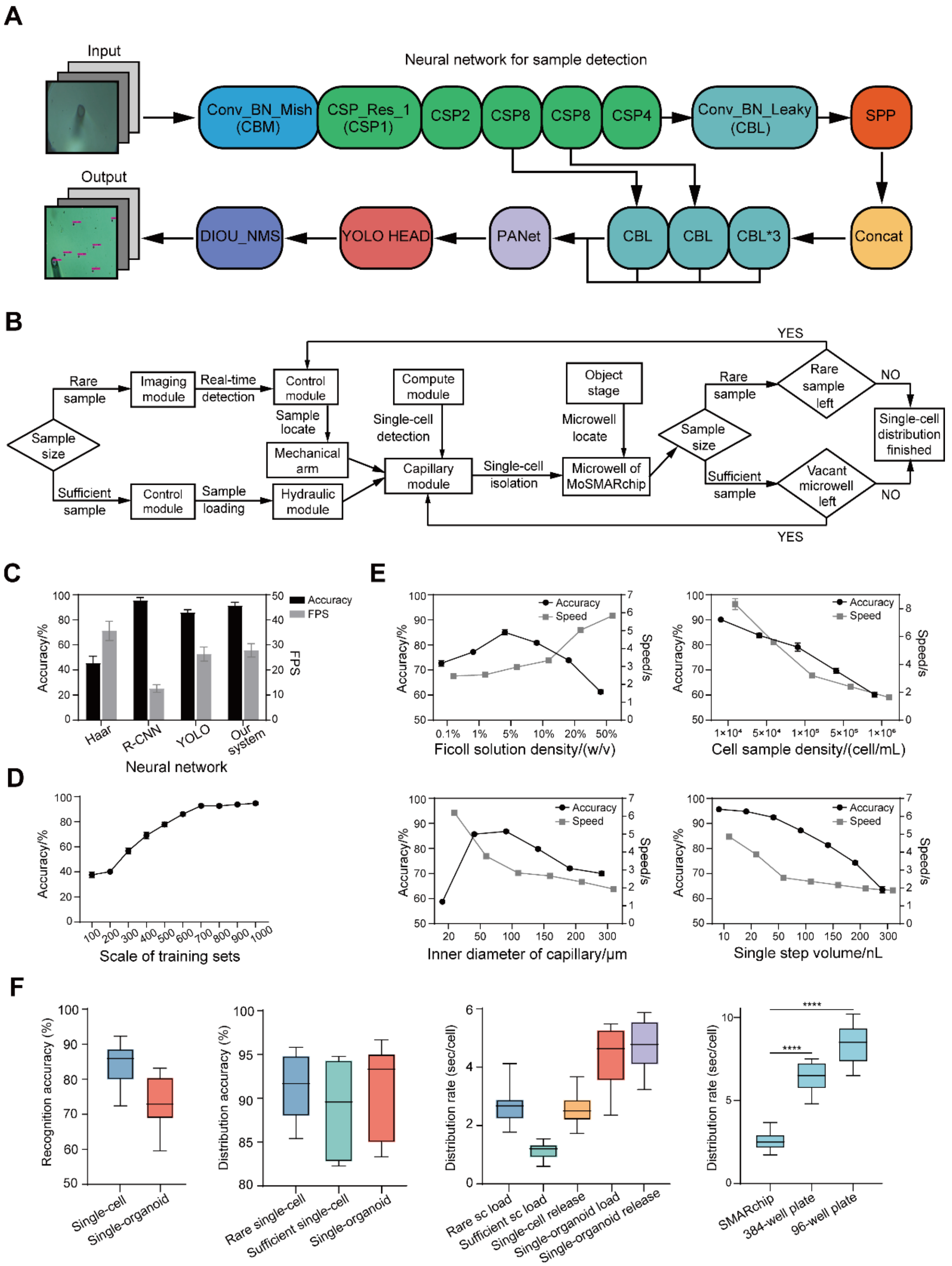

Figure S2. Supplementary illustration and optimization of the automated single cell/organoid distribution instrument. A) The major composition of YOLOv4-based neural network classifier adopted in our system. **B)** The operation logic map of automated single cell distribution instrument. **C-E)** Optimization of the automated single-cell distribution instrument

addressed with major influence factors, including neural network type **(C)**, scale of neural network training set **(D)**, Ficoll density for cell buffer, cell sample density, inner diameter of capillary, and single step accuracy of hydraulic control **(E)**. **F)** Quantification of samples recognition accuracy, distribution accuracy, distribution rate under various circumstances, and comparison of the single-cell distribution rates on the MoSMAR-chip and multi-well plates.

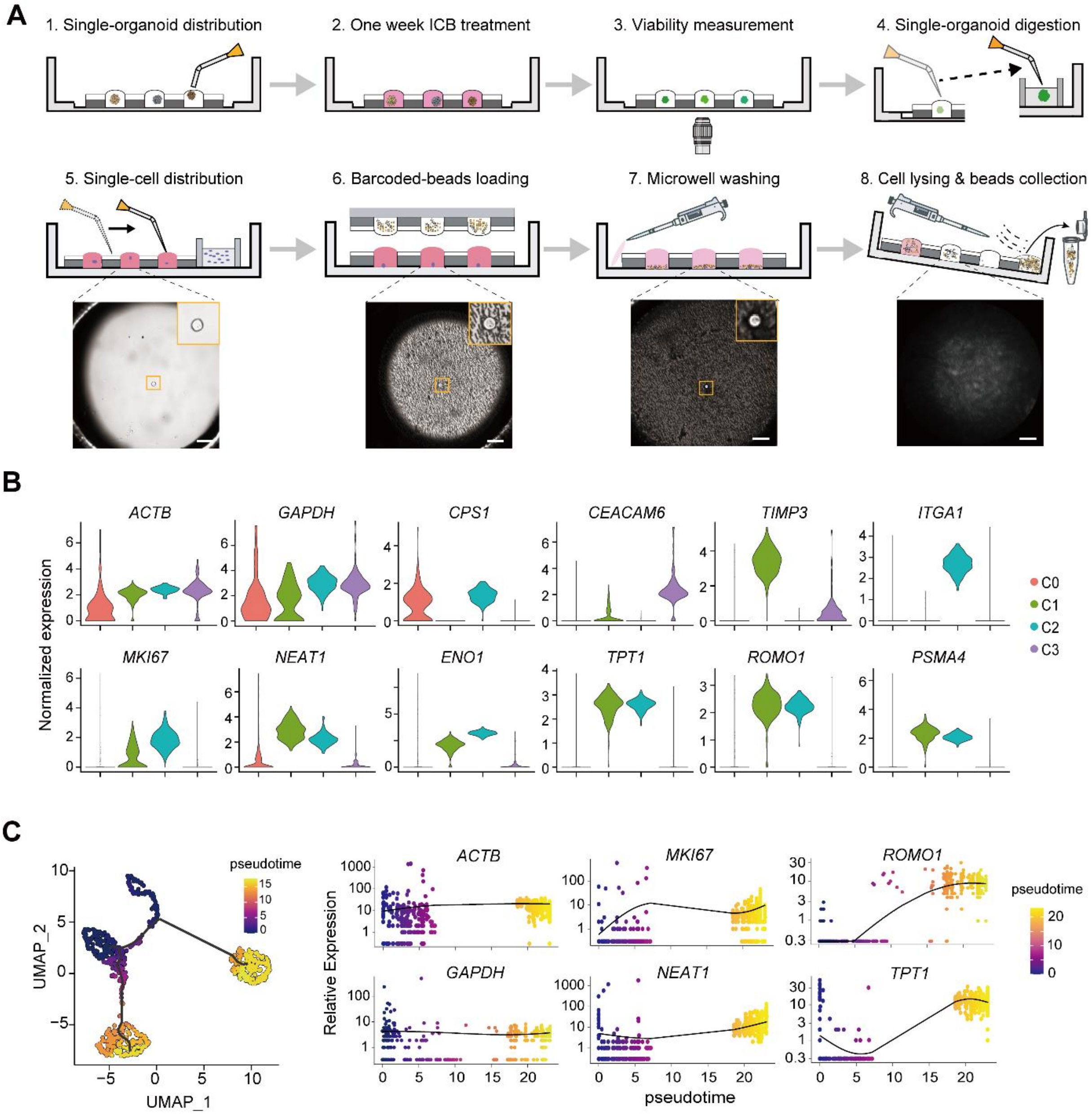

Figure S3. Extended data of Figure 2. **A)** Scheme of the FascRNA-seq procedure for single organoid and images showing single cells in the microwells at the indicated steps. Scale bars: 50 μm. **B)** Violin plots of gene expression features of the four unsupervised clusters in Figure 2J. **C)** Pseudo-trajectory and gene expression analysis along the inferred trajectory.

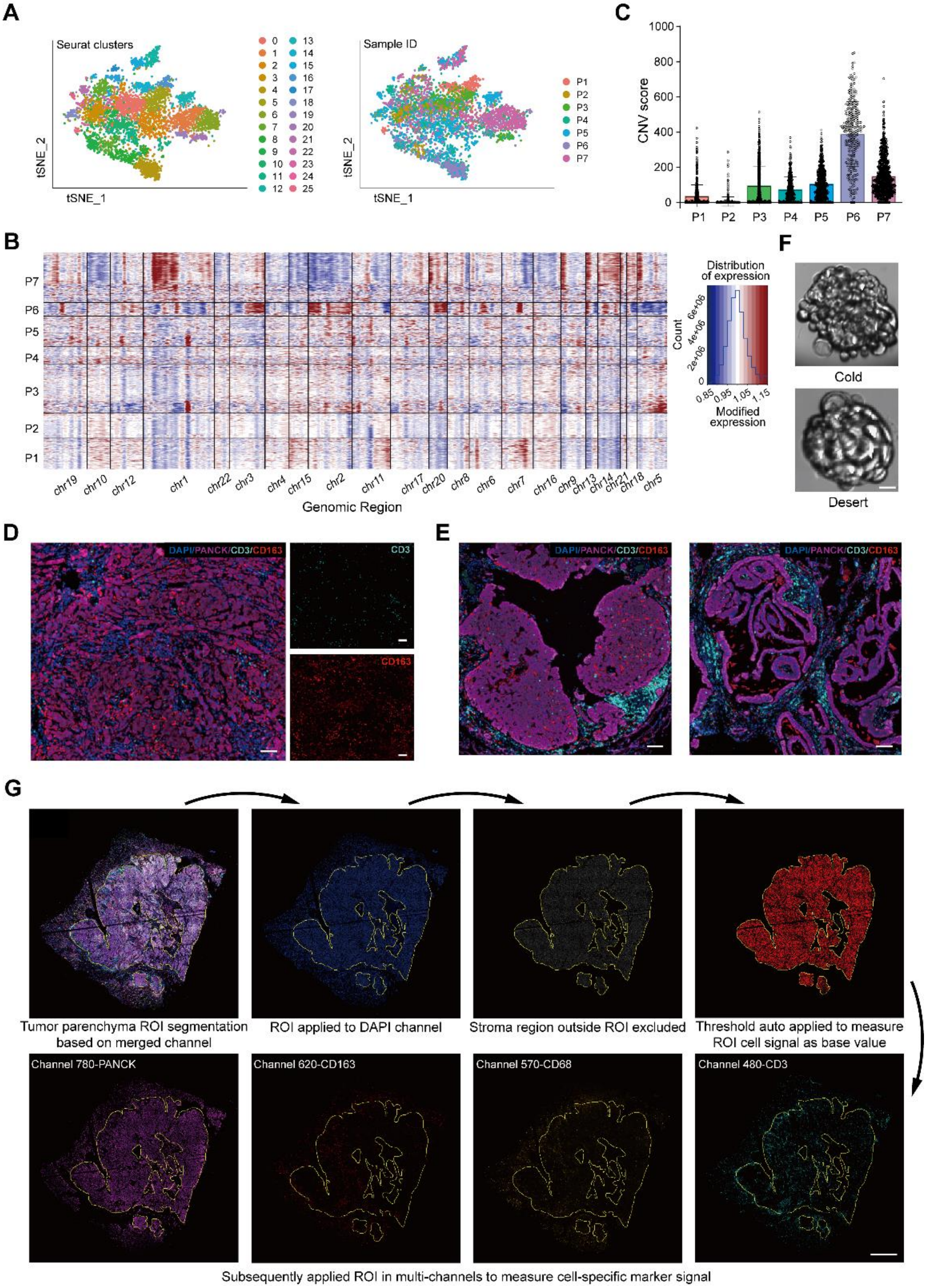

Figure S4. Extended data of Figure 3. A) tSNE visualization of single-cell landscape derived from 171 pLCOs, color labeled by unsupervised seurat clusters (left) or sample IDs (right). **B)** Heatmap of the CNV patterns of all epithelial cells. Red means amplification and blue indicates

deletion. The line chart on the right shows the distribution characteristics of CNV circumstances. **C)** Quantitative assessment of the CNV scores of all epithelial cells in pLCOs derived from seven patient samples. **D and E)** mIHC images of tumor tissue sections of P1 **(D)** and P4 **(E)** with antibodies specific to T cells (CD3), macrophages (CD163), and epithelial cells (PanCK). Cell nucleus was labeled with DAPI. Scale bars: 100 μm. **F)** Representative images of "cold" (left) and "desert" (right) pLCOs derived from P4. Scale bar: 20 μm. **G)** Workflow of mIHC image quantification for the signals of specific cell types in tumor parenchyma. Scale bar: 1 cm.

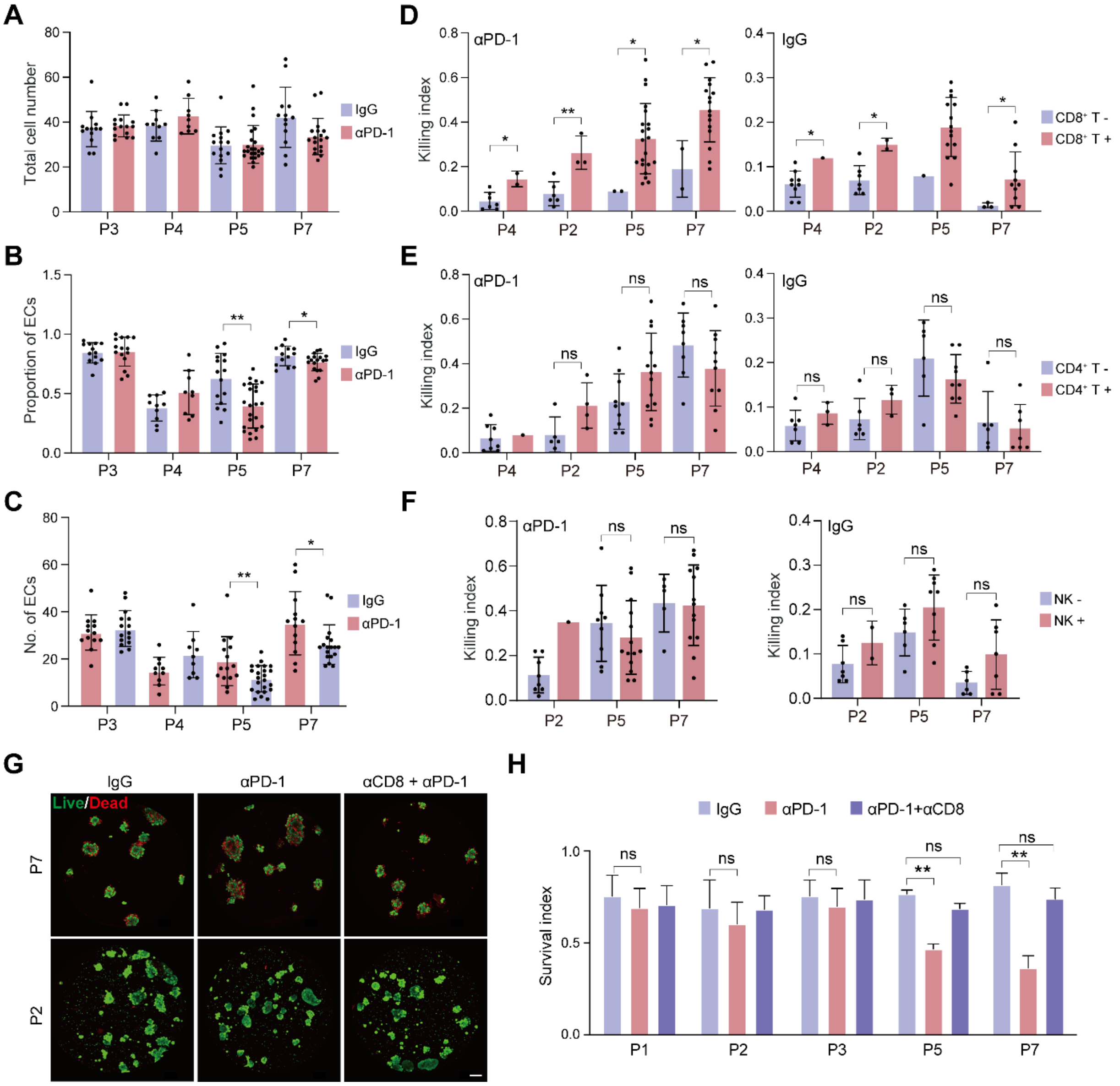

Figure S5. Supplementary data demonstrating CD8$^+$ T cells mediate the αPD-1 induced tumor cell death. A) Comparison of total cell numbers in individual pLCOs with or without αPD-1 treatment. Data from αPD-1 sensitive samples (P5 and P7) and resistant samples (P3, P4) are shown. **B and C)** Comparisons of the numbers **(B)** and proportions **(C)** of epithelial cells in single pLCOs with or without αPD-1 treatment demonstrate the significant reduction of epithelial cells in P5 and P7 pLCOs (n = 116). **D,E and F)** Column bar graphs showing the impact of CD8$^+$ T cells **(D)**, CD4$^+$ T cells **(E)** (n = 106), and NK cells **(F)** on αPD-1 induced cell death (n = 87). **G)** Images of P2 and P7 pLCOs under different treatment conditions. Organoids were stained with Calcein-AM/PI to indicate the living and dead cells. Scale bar: 100 μm. **H)** Comparison of the overall organoid viability under different treatment conditions. Note anti-CD8 (αCD8) antibody treatment rescue the cells from αPD-1 induced cell death (paired student's t tests, n = 3).

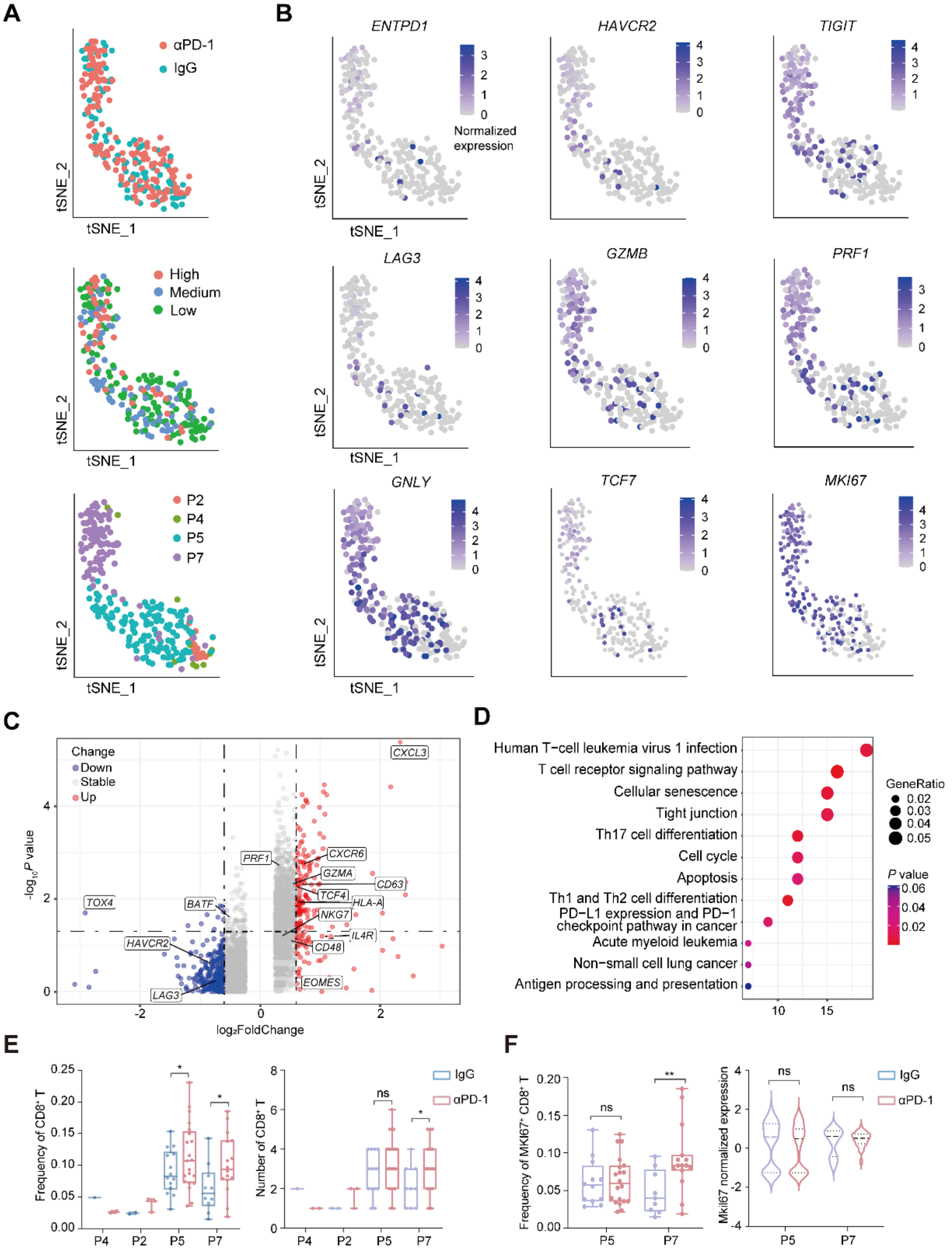

Figure S6. Characterization of parenchyma infiltrating CD8^{+} T cells (PITs) derived from pLCOs. A) tSNE visualization of 191 CD8^{+} T cells from all pLCOs. Data point of each cell was labeled by treatment conditions (top), patient (bottom) and the categories of killing index (Ki) (middle, High: Ki ≥ 0.5; Medium: 0.25 < Ki < 0.5; Low: Ki ≤ 0.25). **B)** Featureplots of 191 T cells derived from pLCOs color labeled with expression levels of T cell function-related

genes. **C)** Volcano plot showing DEGs (P value < 0.05 and fold change ≥ 1.2) between IgG and αPD-1 treated $CD8^+$ T cells. **D)** GO enrichment analysis of the DEGs in **(C)**. **E)** Box plots showing the significant increases in $CD8^+$ T cells in individual pLCOs with the αPD-1 treatment compared to IgG (n = 106). **F)** Comparison of *MKI67* expression in $CD8^+$ T cells under the two treatment conditions (n = 69).

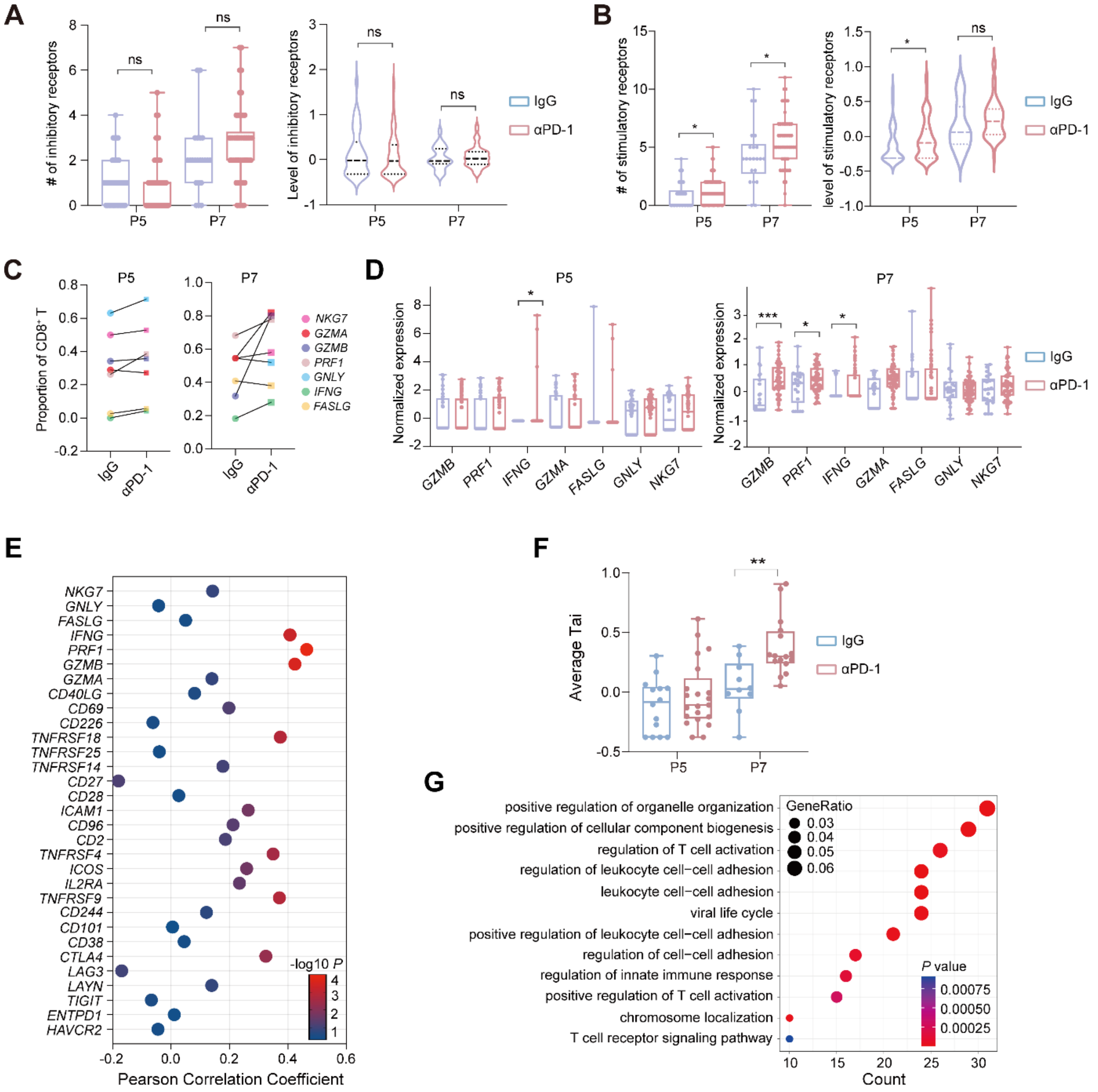

Figure S7. αPD-1 induced gene expression of T cell activation from pLCOs. A and B) Expression of the inhibitory receptors (*HAVCR2, ENTPD1, TIGIT, LAYN, LAG3, CTLA4, CD38, CD101* and *CD244*) and the co-stimulatory receptors (*TNFRSF9, IL2RA, ICOS, TNFRSF4, CD2, CD96, ICAM1, CD28, CD27, TNFRSF14, TNFRSF25, TNFRSF18, CD226, CD69,* and *CD40LG*) in CD8$^+$ T cells under the two treatment groups (n = 69). **C)** Proportions of CD8$^+$ T cells expressing the indicated effector molecules in P5 and P7 pLCOs. **D)** Comparison of the expression levels of effector molecules in CD8$^+$ T cells under the two treatment conditions. **E)** PCC analysis between the average expression levels of function-related genes for CD8$^+$ T cells in pLCOs and the killing index. 10 genes with good correlation were chosen as a geneset to calculate T cell activation index (Tai). **F)** Comparison of average Tai in P5 and P7 pLCOs under two treatment conditions (n = 69). **G)** GO enrichment analysis of the DEGs between the Tai high (>0.5) and other CD8$^+$ T cells.

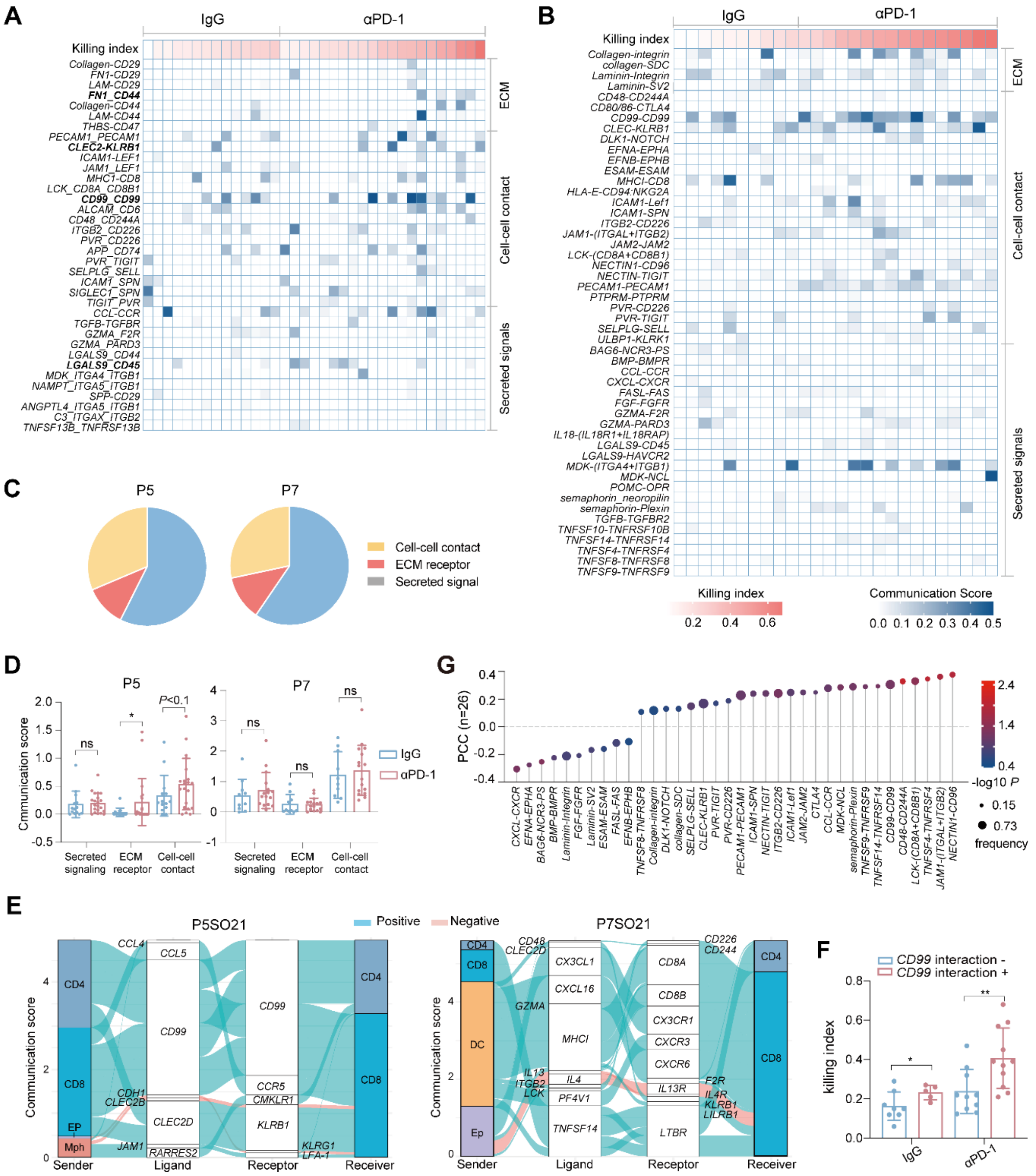

Figure S8. Cell-cell interactions received by CD8+ T cells in individual pLCOs. A and B) Heatmaps showing the cell-cell interactions in individual pLCOs received by CD8+ T cells in P5 **(A)** and P7 **(B)**. The heatmaps are color labeled by the possibility of interactions (i.e., communication score) calculated by CellChat. The bars on the top represent the killing index of individual organoids. **C)** Pie charts showing the abundance of the three types of interactions. **D)** Comparison of the communication scores in individual organoids under the two treatment conditions from P5 (n = 38) and P7 (n = 31). **E)** Sankey plots of intercellular communications between various cell types and T cells in P5SO21 and P7SO21 organoids. The color label represents the positive or negative related ligand-receptor interactions in tumor immunity. **F)** T

cells receiving *CD99* homophilic interaction responded to αPD-1 more significantly. **G)** PCC between communication scores of ligand-receptor pairs and corresponding killing index of P7 pLCOs.

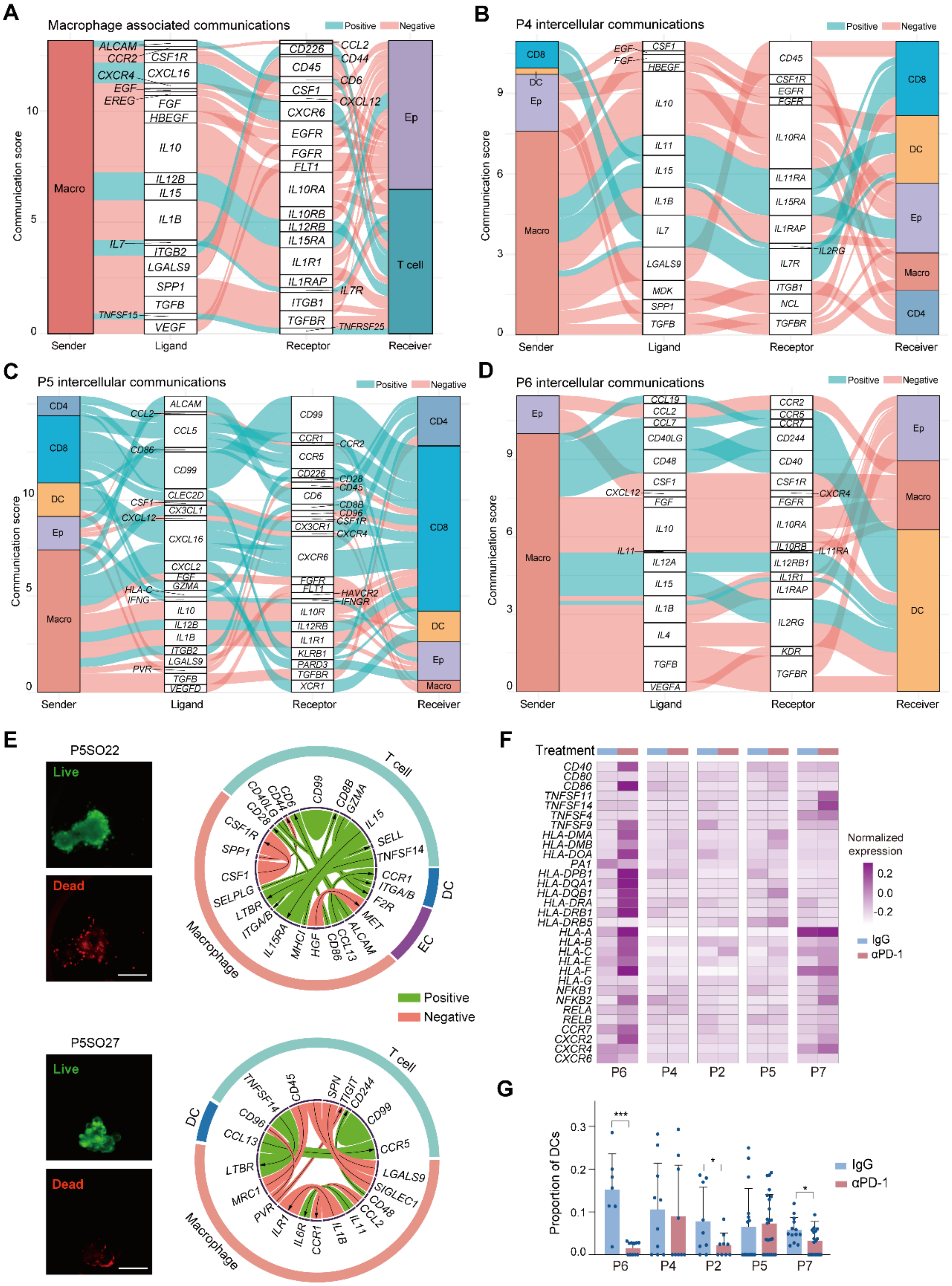

Figure S9. Specific features of TIME for individual patients. A) Sankey plot showing cellular interaction between Mphs and ECs or T cells. Cells from all the P4 and P5 organoids were pooled together for the analysis. **B,C and D)** Sankey plots of intercellular communication landscapes in P4 **(B)**, P5 **(C)**, and P6 **(D)**. Cells from the same tumor sample were pooled

together for the analysis. **E)** Images and interactive circus plots of organoids P5SO22 and P5SO27. Scale bars: 100 μm. **F)** Heatmap showing the average of the normalized expression of DC maturation related genes under the two treatment conditions. **G)** Comparison of DC proportion in individual organoids under the two treatment conditions (*n* = 124).

Supplementary Table Legends

Table S1. Top 40 GO terms most significantly enriched in C3 of P5

Table S2. Pathological information of lung cancer samples

Table S3. The composition of the LCOM medium

Table S4. Reagents and kits used for fresh tumor/ tumor organoids treatment

Table S5. Materials of home-made superhydrophobic paint

Table S6. Components of automated single-cell distribution instrument

Table S7. Reagents and kits used in FascRNA-seq

Table S8. The primers and barcode sequences of FascRNA-seq

Description for Movies S1 to S3

Movie S1. The process of single-cell loading and distribution based on SCDI.

Movie S2. The process of single-organoid loading and distribution based on SCDI.

Movie S3. The comparison of SCDI operation on MoSMAR-chip and a 384well-plate.
